# Microbial Upcycling of Waste PET to Adipic Acid

**DOI:** 10.1021/acscentsci.3c00414

**Published:** 2023-11-01

**Authors:** Marcos Valenzuela-Ortega, Jack T. Suitor, Mirren F. M. White, Trevor Hinchcliffe, Stephen Wallace

**Affiliations:** † Institute of Quantitative Biology, Biochemistry and Biotechnology, School of Biological Sciences, University of Edinburgh, Alexander Crum Brown Road, King’s Buildings, Edinburgh, EH9 3FF, U.K.; ‡ Impact Solutions Ltd., Impact Technology Centre, Fraser Road, Kirkton Campus, Livingston, EH54 7BU, U.K.

## Abstract

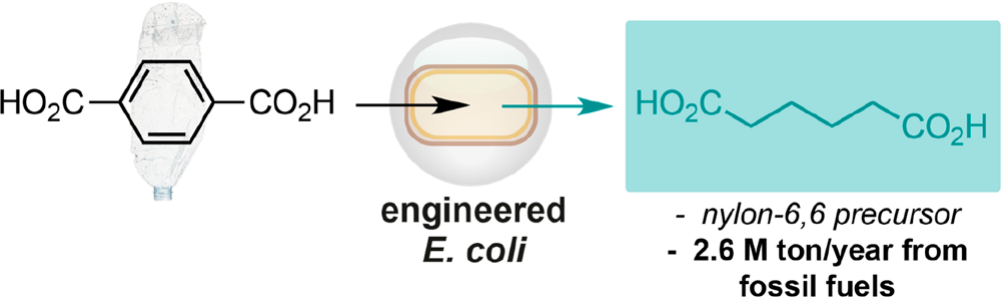

Microorganisms can be genetically engineered to transform abundant waste feedstocks into value-added small molecules that would otherwise be manufactured from diminishing fossil resources. Herein, we report the first one-pot bio-upcycling of PET plastic waste into the prolific platform petrochemical and nylon precursor adipic acid in the bacterium *Escherichia coli*. Optimizing heterologous gene expression and enzyme activity enabled increased flux through the *de novo* pathway, and immobilization of whole cells in alginate hydrogels increased the stability of the rate-limiting enoate reductase BcER. The pathway enzymes were also interfaced with hydrogen gas generated by engineered *E. coli* DD-2 in combination with a biocompatible Pd catalyst to enable adipic acid synthesis from metabolic *cis*,*cis*-muconic acid. Together, these optimizations resulted in a one-pot conversion to adipic acid from terephthalic acid, including terephthalate samples isolated from industrial PET waste and a post-consumer plastic bottle.

## Abstract 2

A *de novo* biosynthesis pathway is reported in the bacterium *Escherichia coli* to convert terephthalate waste from post-consumer and industrial PET samples into the industrial chemical and nylon precursor adipic acid.

## Introduction

Synthetic pathways to industrial chemicals can be designed and assembled in living cells using modern synthetic biology. This enables the bioproduction of target compounds from renewable resources via fermentation and is emerging as an elegant and viable alternative to multistep synthesis from diminishing fossil fuels.^1,2^ Many of these pathways proceed via the fermentation of carbohydrate feedstocks via primary metabolic reactions *in vivo*. However, this approach also enables the upcycling of waste carbon from existing industrial processes to create circular economies, avoiding the environmental consequences of landfill and/or incineration processes. This includes the upcycling of plastic-waste-derived small molecules from post-consumer polyethylene terephthalate (PET)—a thermoplastic material used throughout the modern chemical industry to create a wealth of everyday products. The global demand for this material exceeds 30 M ton/year, of which >80% is designed to be single use, leading to ca. 25 M ton/year of post-consumer PET waste and contributing to the global plastic waste crisis.^3,4^

Although chemical and biological approaches to the depolymerization and recycling of PET waste are being investigated, bio-upcycling technologies to convert plastic waste into higher value small molecules are less established.^5−12^ This approach is attractive as the PET depolymerization products ethylene glycol and terephthalic acid (TA) are microbial metabolites and therefore viable substrates for *de novo* metabolic pathway design. To this end, Kim et al. previously reported the bioconversion of PET-derived ethylene glycol into glycolic acid in *Gluconobacter oxydans* and TA into vanillic acid, muconic acid, gallic acid, and pyrogallol in engineered *E. coli* MG1655 in 33–93% yield.^13^ More recently, Werner et al. reported the high-level bioproduction of β-ketoadipate from *bis*(2-hydroxyethyl)terephthalate (BHET) in 76% yield in engineered *Pseudomonas putida* KT2440.^14^ This was also achieved by Sullivan et al. in 73% yield using *P. putida* AW307 and benzoate isolated from chemically modified mixed plastic waste.^15^ In 2021, our lab reported the conversion of post-consumer PET from a waste plastic bottle into the vanilla flavor compound vanillin in 79% yield in engineered *E. coli* MG1655 RARE.^16^

Following on from this work, we sought to expand the range of small molecules that can be accessed via microbial synthesis from terephthalic acid. Adipic acid (AA) is an aliphatic 1,6-dicarboxylic acid and prolific platform chemical that is used throughout the materials, pharmaceuticals, fragrances, and cosmetics industries. It is currently manufactured on a 2.6 M ton/year scale from petrochemically derived benzene via the nitric acid-catalyzed oxidation of cyclohexanol and cyclohexanone. The process is highly energy intensive and releases a mol/mol equivalent of nitrous oxide into the atmosphere. These emissions have been shown to significantly contribute to global greenhouse gas levels;^17^ 1 kg of N_2_O equates to 298 kg CO_2_ equivalents. As a result, the bioproduction of adipic acid from renewable feedstocks has been an active area of research.

**Figure 1:**
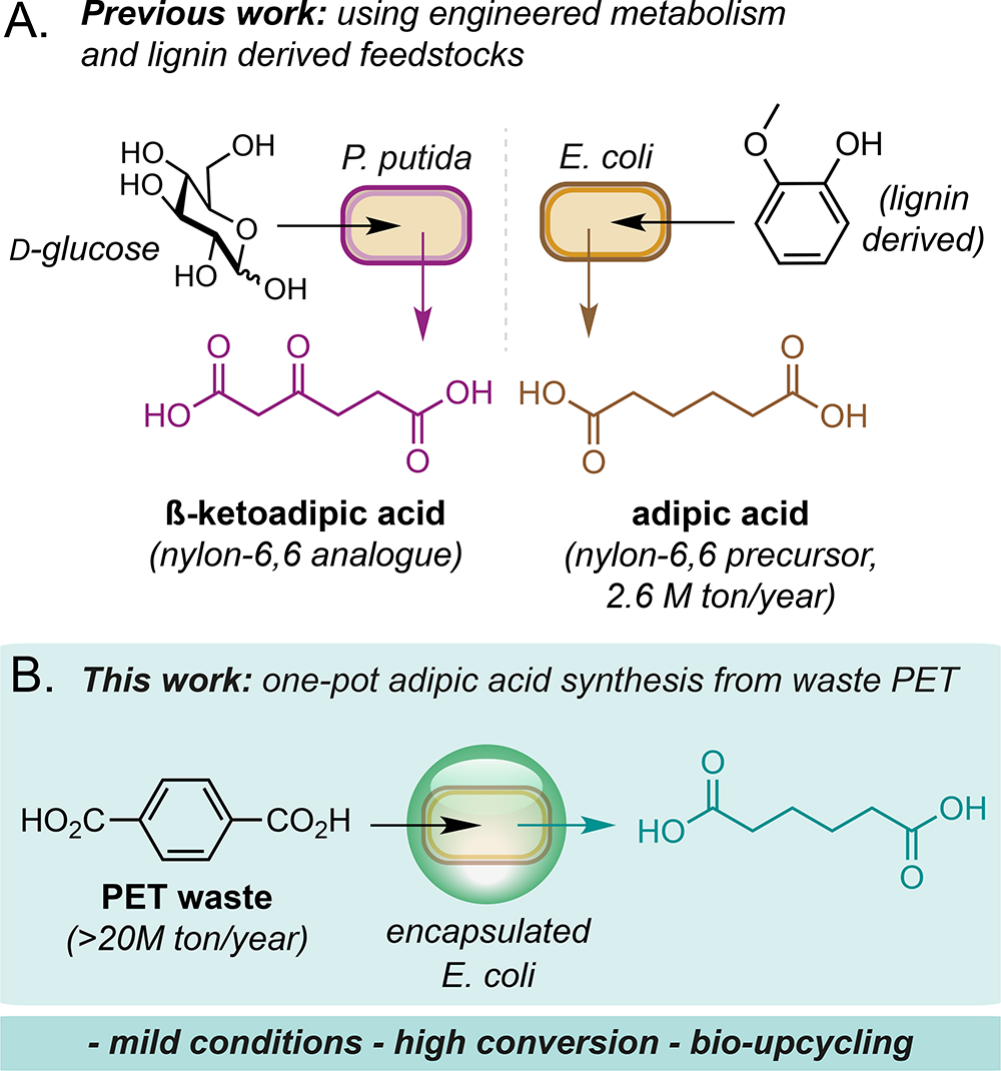
Microbial biotransformation and fermentation approaches to adipic acid and adipate analogues. (A) Carbohydrate fermentation in *P. putida* and valorization of lignin aromatics in *E. coli*. (B) Proposed bio-upcycling of terephthalic acid to adipic acid.

Recent work has included the high-level production of the adipate analogue β-ketoadipate from d-glucose by Rorrer et al. in engineered *P. putida* KT2440 and the one-pot bioconversion of lignin-derived guaiacol to adipic acid by our laboratory in engineered *Escherichia coli* (Figure 1A).^18,19^ However, the microbial synthesis of adipic acid directly from waste PET remains an outstanding challenge in the field of chemical biotechnology. Herein we report the first one-pot bioproduction of adipic acid from terephthalic acid and terephthalate waste in engineered *Escherichia coli*. The reaction proceeds in aqueous media at room temperature and produces adipic acid in 79% conversion (115 mg/L) in 24 h when cells are immobilized in alginate hydrogels (Figure 1B). Together, this study validates the use of microbial cells as a viable biotechnology for the upcycling of plastic-derived small molecules and PET plastic waste.

## Results and Discussion

**Figure 2:**
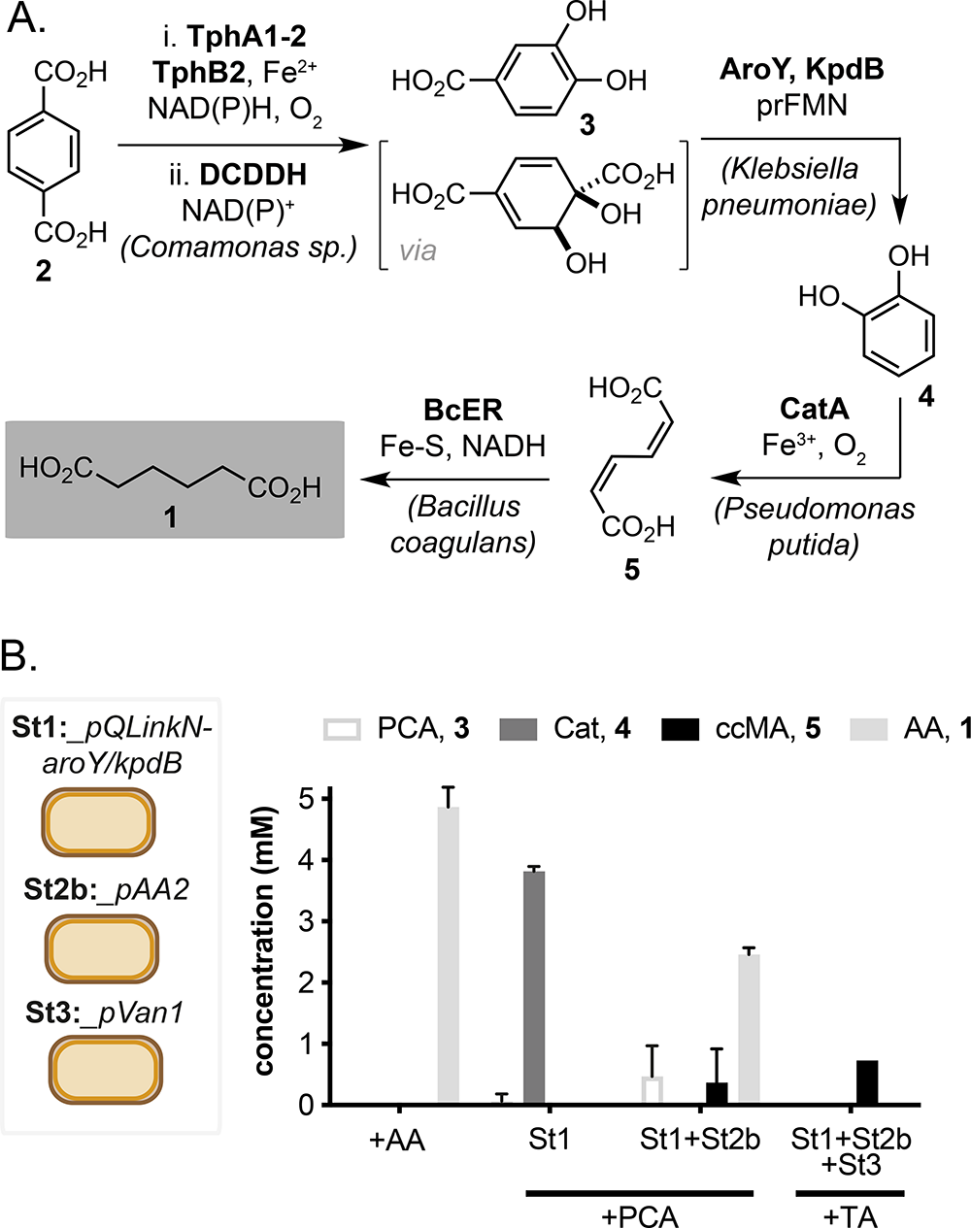
Initial pathway construction and whole-cell activity. (A) The *de novo* biosynthesis pathway to adipic acid from terephthalic acid. (B) Whole-cell mixing experiment. Microbial biocatalysis reactions were performed at OD_600_ 120 at 21 °C in sealed Hungate tubes for 24 h. Product concentrations were determined by reverse-phase HPLC relative to an internal standard of caffeine. All data shown are an average of three replicate experiments to one standard deviation. St1, *E. coli* BL21(DE3)_pQlinkN-*aroY*-*kpdB*; St2b, *E. coli* BL21(DE3)_pAA2(pQlinkN-*catA*-*bcER*); St3, *E. coli* BL21(DE3)_pVan1(*tpado-dcddh*).

We began by assembling the eight genes required for adipic acid synthesis from terephthalate in *E. coli* (Figure 2A, Table S2). The heterologous pathway begins with two enzymes from *Comamonas sp.*: TPADO, a heterotrimeric O_2_-dependent dioxygenase consisting of TphA1–2 and TphB2 subunits, and DCDDH, a NAD^+^-dependent dehydrogenase. Together, these enzymes catalyze the oxidative decarboxylation of terephthalate to protocatechuate (PCA). Protocatechuate is then transformed into adipic acid by four additional enzymes: AroY, KpdB, CatA, and BcER. AroY is a protocatechuate decarboxylase from *Klebsiella pneumoniae*; KpdB is the B-subunit of 4-hydroxybenzoate decarboxylase from *K. pneumoniae* that activates AroY by generating prenylated FMN (prFMN); CatA is a non-heme Fe(III)-dependent dioxygenase from *Pseudomonas putida*; and BcER is a [4Fe–4S]-dependent oxidoreductase from *Bacillus coagulans*.^20^

The *aroY* and *kpdB* genes were inserted into an empty pQLinkN backbone (Figure S1), and the plasmid was transformed into *E. coli* BL21(DE3). Similarly, our previously reported pAA and pAA2 plasmids (pETDuet-1 and pQLinkN encoding *catA* and *bcER*, respectively^19^) and pVan1 plasmid (encoding TPADO and DCDDH^16^) were also transformed into *E. coli* so as to conduct an initial whole-cell mixing experiment. This was to determine whether AA could be detected when PCA or TA was added to suspended whole-cell mixtures of *E. coli* BL21(DE3)_pQLinkN-*aroY*-*kpdB* (termed St1), *E. coli* BL21(DE3)_pAA (termed St2), *E. coli* BL21(DE3)_pAA2 (termed St2b), and *E. coli* BL21(DE3)_pVan1 (termed St3; Figure 2B and Figure S9).

To this end, cells were grown to mid-log phase and protein expression was induced for 24 h. Cells were isolated, resuspended in equal amounts to OD_600_ 120 in M9 media containing 5 mM PCA/TA, and incubated at 21 °C for a further 24 h. Gratifyingly, St1 was able to convert PCA to catechol in 76% conversion. St2 was able to convert catechol to adipic acid in 79% conversion. A co-culture of St1 and St2 collectively harboring AroY, KpdB, CatA, and BcER was able to convert PCA to *cis*,*cis*-muconic acid (ccMA) in 48% conversion by HPLC, with no AA detected. In comparison, a co-culture of St1 and St2b (harboring the pAA2 plasmid) transformed PCA to adipic acid in 49% yield, presumably due to mild T5 as opposed to strong T7-induced expression of BcER from pAA2 *in vivo* (Figure S1 and Table S1). However, when St1, St2b, and St3 were combined, ccMA was produced as the major product in 19% yield, with the remaining material being unreacted TA—indicating both TPADO and BcER activity as pathway bottlenecks.

**Figure 3:**
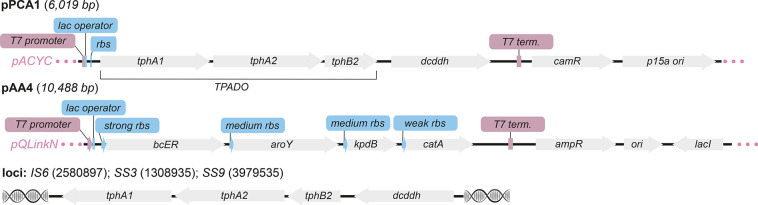
Maps of the pPCA1 and pAA4 plasmids and chromosomal loci for tpado and dcddh integration in *E. coli*.

Having confirmed that AA and ccMA could be produced from PCA and TA, respectively, in a multicell biotransformation, we hypothesized that localization of all the pathway enzymes to a single cell would increase product conversions. We therefore moved on to design genetic constructs that could be used to balance the expression and activity of TPADO and BcER with the aim of producing a single *E. coli* strain to produce AA from TA. To this end, the expression cassette encoding the *tphA1–2*/*B2* and *dcddh* genes was transferred from our reported pVan1 plasmid and ligated into a pACYC-derived backbone, creating pPCA1 (Figure 3 and Figure S2). The pACYC vector is a medium/low copy-number plasmid with a p15A origin-of-replication and Cam^R^ selection marker that are compatible with the pQLinkN vector harboring the remaining pathway genes. The *aroY* and *kpdB* genes were inserted into pAA2 by ligation-independent cloning to generate pAA3 (Figure S1). Plasmids pPCA1 and pAA3 were then transformed into *E. coli* BL21(DE3), and a whole-cell biotransformation was conducted to determine whether adipic acid could be detected when TA was added to suspended whole cells. Unfortunately, no AA was observed from these reactions by HPLC. The major product was ccMA in 92% yield, reconfirming that the expression and/or activity of BcER was a limiting step in the pathway. The increased yield of ccMA, however, validated that single cell co-expression of the pathway enzymes was sufficient to increase product flux.

We therefore designed a revised pAA3 plasmid (pAA4, Figure 3 and Figure S2) to express the *aroY*, *kpdB*, *catA*, and *bcER* genes as part of a polycistronic mRNA. Here, *bcER* was assembled at the 5′ end of the operon and contained a strong ribosomal binding site (RBS), followed by *aroY* (medium RBS), *kpdB* (medium RBS), and *catA* (weak RBS) (Figure 3). The latter is the most active enzyme in the pathway and therefore required minimum expression for maximum product flux. We also hypothesized that this arrangement would maximize expression of *bcER* and therefore enable increased conversion of ccMA to AA from TA. We also integrated the PCA1 cassette into the genome of *E. coli* BL21(DE3) via λ-Red recombineering using CRISPR/Cas9 as a negative selection tool,^20^ with the aim of decreasing the overall metabolic burden to the host cell (Figure 3). IS6, SS3, and SS9 loci were selected in the *E. coli* BL21(DE3) genome (positions 2580897, 1308935, and 3979535, respectively) as equivalent sites in *E. coli* BW25113 were originally reported to be suitable for genomic integration,^21^ generating the strains *E. coli* IS6::PCA1, *E. coli* SS3::PCA1, and *E. coli* SS9::PCA1. Successful knock-ins were confirmed by colony PCR using primers that bind to genomic regions flanking the corresponding insertion loci. The pAA4 plasmid was transformed into these strains, or co-transformed into *E. coli* BL21(DE3) with pPCA1 and used in a whole-cell biotransformation experiment. Singly transformed PCA1 integrants were also co-transformed with pACYC plasmids encoding for the molecular chaperones DnaK-DnaJ-GrpE (pKJE7), GroEL-GroES (pGro7), and Trigger Factor (pTf16) to facilitate soluble folding of the heterologous pathway enzymes.

**Figure 4:**
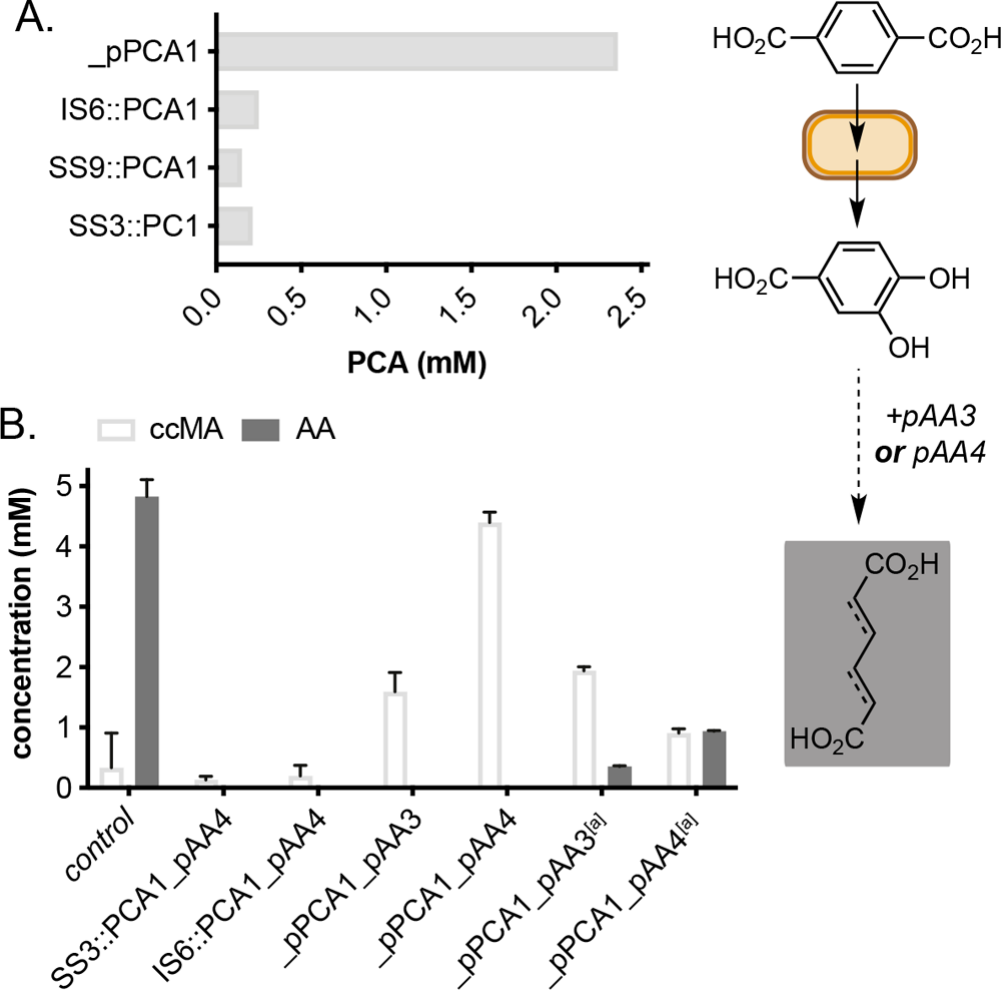
Comparing the reactivity of PCA1 plasmid and genome integration strains. (A) Whole-cell reactions to protocatechuate. (B) Whole-cell reactions to muconic acid and adipic acid. Product concentrations were determined by reverse-phase HPLC relative to an internal standard of caffeine. All data shown are an average of three replicate experiments to one standard deviation. [a] ccMA was added instead of TA.

Unfortunately, no adipic acid was produced from *E. coli* BL21(DE3)_pPCA1_pAA4 cells after 24 h at 21 °C. Adipic acid was detected in 19% yield from this strain when cells were fed ccMA (Figure 4B, Figures S11 and S12). The genome integration of PCA1 to IS6, SS3 or SS9 loci did not increase adipic acid production and reduced the overall levels of ccMA (Figure 4A,B). Comparison of PCA production from TA in *E. coli* IS6::PCA1, *E. coli* SS3::PCA1, and *E. coli*_pPCA1 confirmed that PCA formation was significantly increased in strains containing pPCA1 (Figure 4A and Figure S9). Chaperone co-expression reduced and/or abolished ccMA levels in all cells expressing pAA4 and PCA1 and did not result in any detectable AA from any PCA1 integrated strains. The PCA and AA expression cassettes from pPCA1 and pAA4 were also swapped (generating plasmids pAA5 and pPCA2; Figure S3) to increase the copy number of *tpado* and *dcddh* genes and increase flux to PCA from TA. However, kinetic analysis of PCA and ccMA production indeed confirmed that PCA was produced more rapidly in *E. coli*_pPCA2_pAA5 from 2 to 4 h but that ccMA titers were ultimately lower in this strain (31% yield) when compared to *E. coli*_pPCA1_pAA4 (93% yield; Figure S14). Due to low TPADO activity and increased ccMA titers using pPCA1 and pAA4, we decided to proceed with this two-plasmid system and to focus our studies on optimization of the whole-cell biotransformation.

We began by assessing AA production from TA by our engineered strains under fermentation conditions, anticipating that addition of TA immediately after synthesis of the pathway enzymes would mitigate the instability of TPADO and/or BcER. As such, TA was added to cultures at OD_600_ 0.5 and reactions were sampled periodically over 7 days. Interestingly, no AA could be detected from cells transformed with pPCA1 and pAA3 plasmids. Cells transformed with pPCA1 and pAA4 produced adipic acid in 6% yield, and *E. coli* IS6::PCA1_pAA4 and *E. coli* IS6::PCA1_pAA4_pGro7 strains produced AA in 6% and 5% yield, respectively. Altering the fermentation growth media or carbon source eliminated AA production from all strains and produced PCA as the primary product.

**Figure 5:**
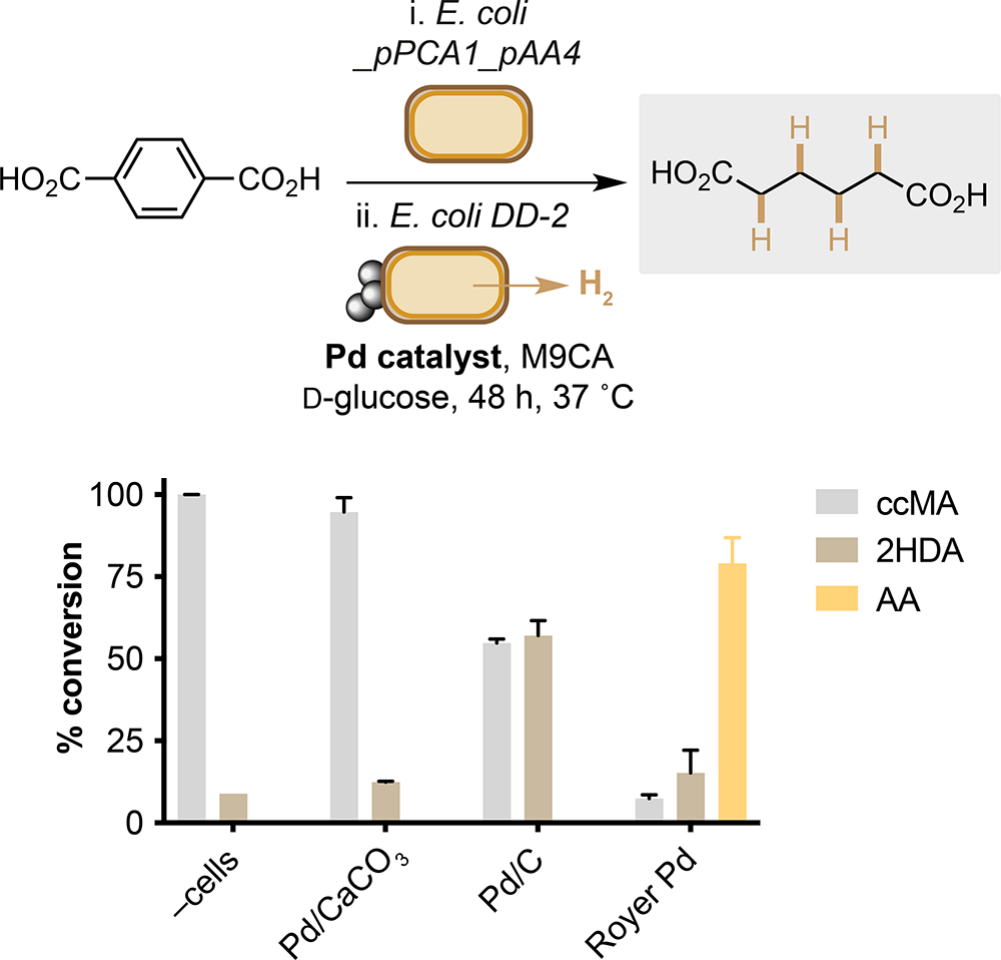
Bio-hydrogenation of metabolic *cis*,*cis*-muconic acid from *E. coli* BL21(DE3)_pPCA1_pAA4, followed by *E. coli* DD-2 and biocompatible Pd catalysts. Product concentrations were determined by reverse-phase HPLC relative to an internal standard of caffeine. Data shown are an average of three replicate experiments. 2HDA = 2-hexenedioic acid.

BcER activity therefore continued to be the rate-limiting step in the pathway, so we progressed to investigating methods to overcome this using chemical approaches (Figures S17–S20). Chemical methods included the use of biocompatible chemistry to replace the activity of BcER by converting ccMA to AA using a H_2_-generating strain of *E. coli* (DD-2) and a membrane-bound Pd catalyst. Microbial H_2_(g) has been shown to reduce ccMA *in vitro* using the Royer Pd catalyst,^22^ but this has not been combined with metabolic ccMA generation. To this end, ccMA was produced from *E. coli*_pPCA1_pAA4 before cells were removed by centrifugation and the supernatant containing ccMA introduced to a culture of *E. coli* DD-2. This engineered strain contains an insulated pathway consisting of a pyruvate ferredoxin oxidoreductase (PFOR) from *Desulfovibrio africanus*, hydrogenase maturation factors from *Chlamydomonas reinhardtii*, and a ferredoxin and [Fe–Fe] hydrogenase from *Clostridium acetobutylicum*, which together enable the anaerobic production of H_2_(g) from d-glucose.^23^ Indeed, this enabled the bio-hydrogenation of metabolic ccMA to AA in 80% yield using the biocompatible Royer Pd catalyst (Figure 5). Intriguingly, both Pd/CaCO_3_ and Pd/C were biocompatible but inactive bio-hydrogenation catalysts, affording 14% and 64% of the monoreduced product 2-hexenedioic acid (2-HDA), respectively. The increased reactivity of Royer Pd is therefore likely due to an attractive electrostatic interaction between the cells, ccMA, and the positively charged polyethyleneimine catalyst support. Hydrogen-gas-producing *E. coli* and biocompatible chemistry can therefore be used to overcome the lack of activity of BcER within larger heterologous biosynthetic pathways.

**Figure 6:**
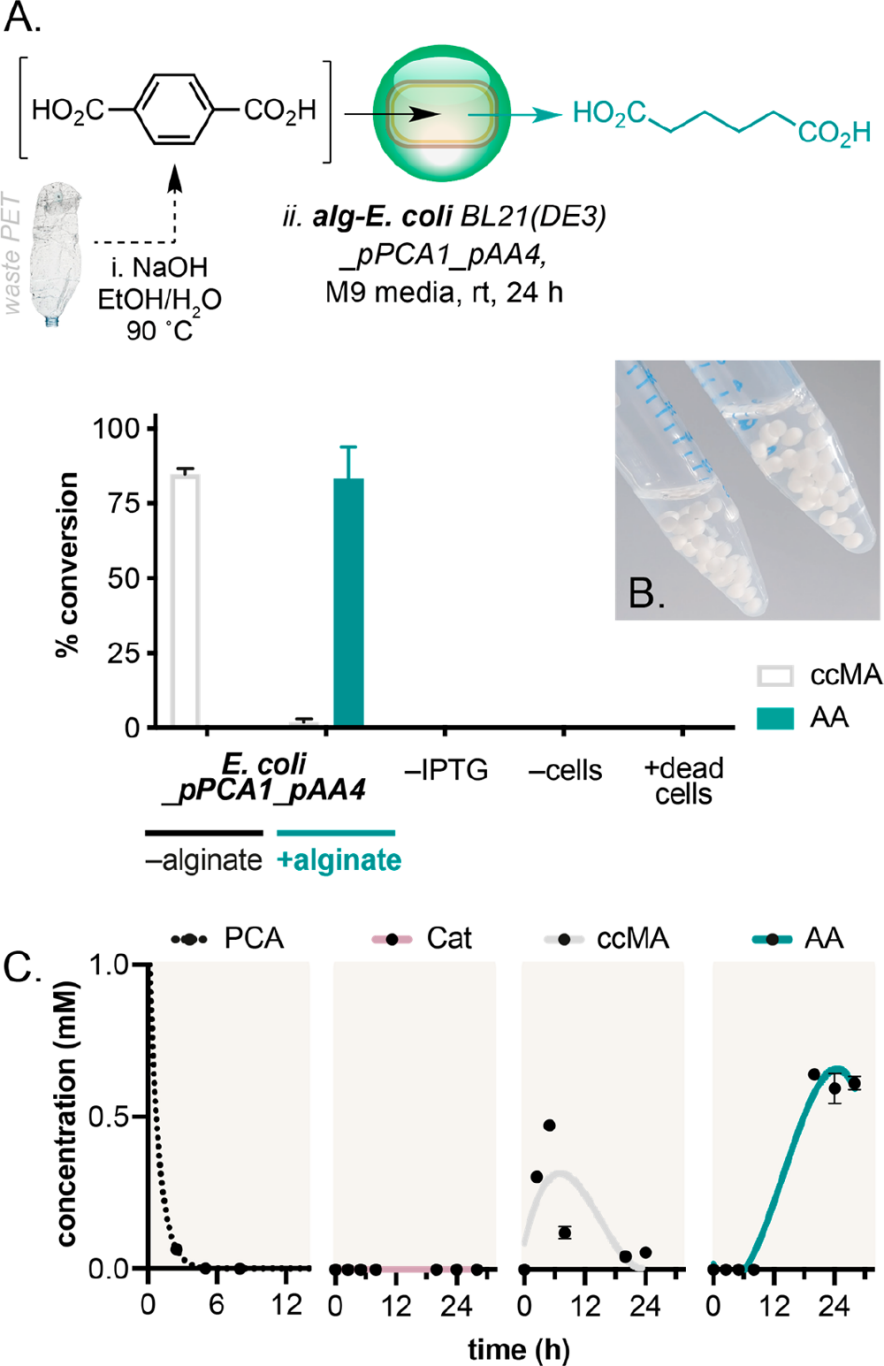
*E. coli* supported in calcium alginate beads enables bio-adipic acid production from a post-consumer plastic bottle. (A) PET depolymerization and upcycling in whole cells and in encapsulated whole cells. Reactions were carried out at OD_600_ 60 in M9 media with 166 mg/L TA at 21 °C with shaking at 220 rpm for 24 h. (B) Photograph of alg-*E. coli* cells. (C) Metabolite formation during biotransformation reactions. Product concentrations were determined by reverse-phase HPLC relative to an internal standard of caffeine. Data shown are an average of three replicate experiments to one standard deviation.

Finally, we examined the use of cells supported in alginate hydrogel as a method to increase the activities of TPADO and BcER and thus the yield of AA. The use of calcified alginate hydrogels is known to increase the stability of enzymes *in vitro*(24,25) and to improve the downstream purification of whole-cell biotransformations; however, few studies report the use of alginate immobilization to increase the stability of heterologous enzymes in *de novo* pathways in *E. coli*. More specifically, this has not been applied to stabilizing BcER within a microbial adipic acid pathway despite reported instability *in vivo*.^20,26^ To this end, we were delighted to observe that, when TA was added to cells of *E. coli*_pPCA1_pAA4 supported in alginate hydrogels (termed alg-*E. coli*), AA conversion was increased from 0% to 79% (Figure 6B). Adipic acid was not detected in control samples lacking cells, alginate, and/or pathway enzymes or in the presence of supported dead cells, confirming that this was a microbe-mediated chemical transformation. A time-course experiment confirmed that alginate immobilization increased the stability of BcER, showing rapid formation of 2-hexenedioic acid from ccMA after 6 h and then gradual conversion to AA over 24 h (Figure 6C). In comparison, no reduction of ccMA to AA was observed after 24 h in the absence of the alginate support. Finally, adipate-rich product streams could be readily isolated from alg-*E. coli* reactions by filtration of the cell-containing alginate beads from the reaction. Muconic acid reduction in reactions with non-immobilized cells or in alg-*E. coli* with smaller bead sizes also produced less adipic acid, indicating that the alginate support likely improves the oxygen tolerance and/or stability of the [4Fe–4S]-containing BcER enzyme. This was confirmed by observing loss of BcER activity in <6 h in the absence of the alginate support (Figure S20)—a time point which precludes the accumulation of ccMA by upstream pathway enzymes in *E. coli*_pPCA1_pAA4 strains.

Following this finding, three factors were explored to improve the activity of the *de novo* pathway: (i) increased cofactor availability and recycling, (ii) pH-dependent TA diffusion into the cell, and (iii) BcER inhibition by upstream intermediates. First, NADH availability was identified as a potential key limitation as the *de novo* pathway to adipic acid from TA generates 3 mol equivalents of NAD^+^ and only regenerates 1 mol equivalent of NADH. We therefore co-expressed the NAD^+^-dependent formate dehydrogenase Fdh from the methylotrophic bacterium *Pseudomonas* sp. 101 (EC 1.17.1.9) downstream of medium or strong constitutive promoters in modified pPCA1 plasmids (pPCAX1–3, Table S1) in immobilized alg-*E. coli*_pPCA*X*_pAA4 strains, generating 1 mol of NADH from 1 mol each of NAD^+^ and formate.^27^ However, *fdh* co-expression resulted in no change in adipic acid formation at increased TA concentrations in either the presence or absence of exogenous formate. Such strategies have been successfully applied to counteract nicotinamide redox imbalance in other metabolically engineered strains.^28^ Reactions containing Fdh and run in the presence of formate also became alkaline over time—conditions that are known to inhibit TA diffusion into the cell by increasing repulsive ionic interactions with the negatively charged outer membrane (p*K*_a__1_ 3.5 and p*K*_a__2_ 4.5 in H_2_O). This was confirmed by observing increased conversion of TA into PCA in *E. coli*_pPCA1_pAA4 cells at pH 5. However, this was accompanied by decreased downstream pathway activity (Figure S22). We therefore moved on to examine the use of increased concentrations of glucose and the use of alternative carbohydrate feedstocks as a source of NADH *in vivo*.^29,30^ Switching the carbon source from d-glucose to d-mannitol or d-sorbitol—two hexose sugar alcohols that generate more NADH equivalents than glucose during glycolysis—had no effect on adipic acid levels, nor did the co-addition of glucose and sorbitol at 1:1 mol equivalent or increasing the concentration of glucose 2-fold (Figure S24). Together, these data combined with the observed increase in adipic acid production from alginate reactions run in diluted media with reduced glucose concentration (Figure 6A,C) make cofactor availability and redox balance an unlikely limiting factor at this scale but nevertheless one that should be a primary consideration in subsequent strain and process designs. Finally, we examined the inhibition of BcER by TA. Interestingly, TA was found to inhibit BcER activity at high substrate concentrations, presumably due to similarities in three-dimensional structure between the *cis*-oid diacid in ccMA and the 1,4-disubstituted aromatic diacid in TA (Figure S22). Inhibition of BcER by TA in combination with pH-dependent TA diffusion and flux at physiological pH are therefore principal considerations that will be the focus of our future work.

**Figure 7:**
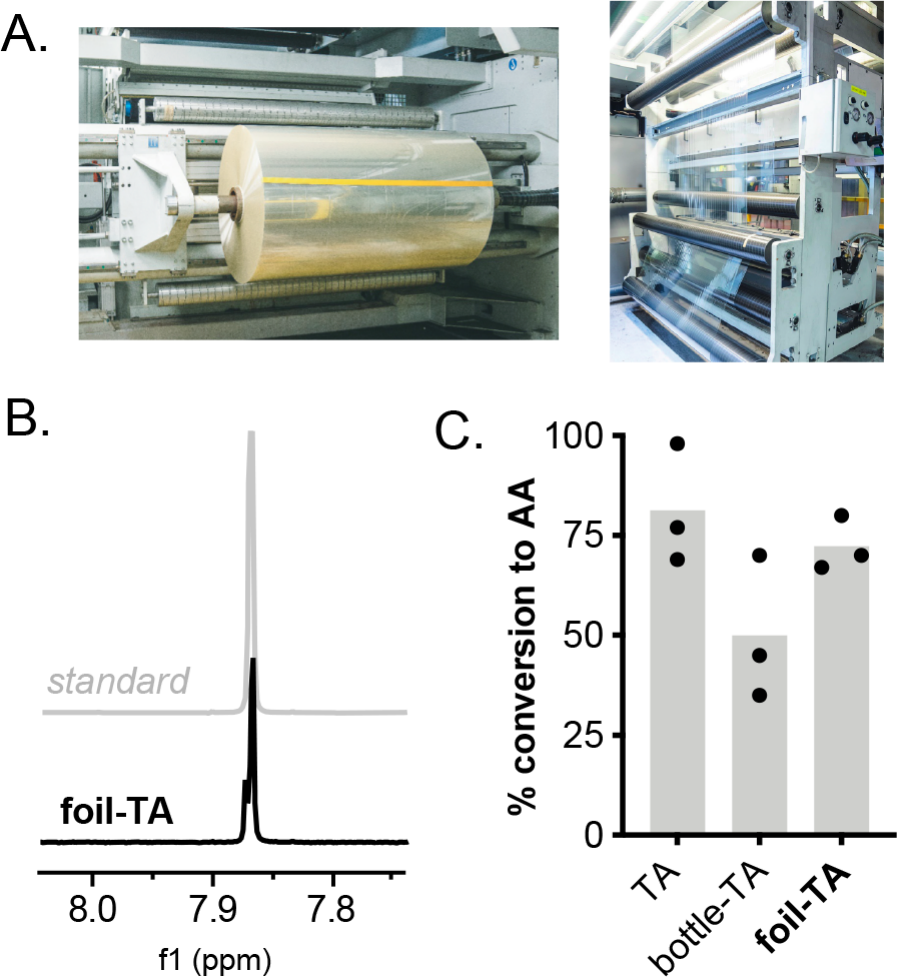
Microbial upcycling of industrial PET stamping foil waste. (A) Image of PET stamping foils. (B) ^1^H NMR spectrum of foil-TA. (C) Bio-upcycling of PET/TA samples into adipic acid. Data shown are an average of three replicate experiments to one standard deviation.

Having confirmed that we can convert TA into AA using engineered *E. coli*, we set out to examine whether alg-*E. coli*_pPCA1_pAA4 could be used to valorize post-consumer plastic waste. To this end, a discarded PET bottle was depolymerized using aq. NaOH and ethanol (90 °C, 1 h), yielding white flakes of pure TA by ^1^H NMR analysis. To our delight, addition of crude TA samples to alg-*E. coli*_pPCA1_pAA4 cells resulted in 65 mg/L AA by HPLC. To further demonstrate the applicability of this system and avoid the compositional variability of PET bottles,^31^ we also examined the use of pure industrial PET waste. Hot stamping foils (HSFs) are used across multiple industries for the rapid depositing of ultrathin release single-use lacquer and adhesive labels. In 2022, the global demand for HSFs was 2.5 billion m^2^, and this is estimated to generate 40,000 tons of PET waste per annum. Pleasingly, depolymerization of HSF samples under identical alkaline hydrolysis conditions (aq. NaOH, EtOH, 90 °C, 1 h) yielded pure TA by ^1^H NMR, which could be converted to AA under our optimized biotransformation conditions in 66% yield (96 mg/L) using alg-*E. coli*_pPCA1_pAA4 cells (Figure 7). This increased conversion establishes HSFs as a source of PET waste that is highly amenable to microbial upcycling processes.

## Conclusions

In summary, the development of new sustainable bio-based methods to valorize waste carbon into industrial small molecules is an elegant approach to creating a circular chemicals economy. Through a series of chemical and genetic optimizations, this study reports the first bioproduction of the prolific platform chemical adipic acid directly from terephthalic acid generated *in situ* from industrial PET waste and a post-consumer plastic bottle. The reaction occurs in engineered *E. coli* cells through an eight-gene, six-enzyme *de novo* biosynthetic pathway within calcified alginate beads. Product conversion is high (79%, 115 mg/L) and occurs in aqueous media under ambient conditions (room temperature, pH 7.4) in 24 h. We believe this is the first report of the bioproduction of adipic acid from a plastic waste source, substantiating the use of microbial biotechnology as a solution to the valorization of this abundant “waste” feedstock while also diverting chemical manufacturing routes away from the sole use of raw petrochemicals. Future work from our lab will include process intensification focused on cofactor recycling and parameters such as terephthalate import, BcER engineering, scale-up, and extension of this pathway to encompass the microbial synthesis of other chemical targets of industrial significance.

## Supporting Information Available

Materials and methods, DNA sequences, plasmid assembly, transformation and genome integration protocols, protein overexpression conditions, whole-cell catalysis and alginate immobilization procedures, PET bottle and stamping foil depolymerization reaction conditions, and terephthalate analysis (PDF)

## References

[ref1] Becker J.; Wittmann C. Advanced Biotechnology: Metabolically Engineered Cells for the Bio-Based Production of Chemicals and Fuels, Materials, and Health-Care Products. Angew. Chem., Int. Ed. 2015, 54 (11), 3328–3350. 10.1002/anie.201409033. 25684732

[ref2] Cho J. S.; Kim G. B.; Eun H.; Moon C. W.; Lee S. Y. Designing Microbial Cell Factories for the Production of Chemicals. JACS Au 2022, 2 (8), 1781–1799. 10.1021/jacsau.2c00344. 36032533 PMC9400054

[ref3] Geyer R.; Jambeck J. R.; Law K. L. Production, use, and fate of all plastics ever made. Science Advances 2017, 3 (7), e1700782 10.1126/sciadv.1700782. 28776036 PMC5517107

[ref4] Plastic upcycling. Nature Catalysis 2019, 2 ( (11), ), 945–946. 10.1038/s41929-019-0391-7.

[ref5] Tiso T.; Narancic T.; Wei R.; Pollet E.; Beagan N.; Schröder K.; Honak A.; Jiang M.; Kenny S. T.; Wierckx N.; et al. Towards bio-upcycling of polyethylene terephthalate. Metabolic Engineering 2021, 66, 167–178. 10.1016/j.ymben.2021.03.011. 33865980

[ref6] Wei R.; Tiso T.; Bertling J.; O’Connor K.; Blank L. M.; Bornscheuer U. T. Possibilities and limitations of biotechnological plastic degradation and recycling. Nature Catalysis 2020, 3 (11), 867–871. 10.1038/s41929-020-00521-w.

[ref7] Zhou H.; Ren Y.; Li Z.; Xu M.; Wang Y.; Ge R.; Kong X.; Zheng L.; Duan H. Electrocatalytic upcycling of polyethylene terephthalate to commodity chemicals and H2 fuel. Nat. Commun. 2021, 12 (1), 4679 10.1038/s41467-021-25048-x. 34404779 PMC8371182

[ref8] Blank L. M.; Narancic T.; Mampel J.; Tiso T.; O’Connor K. Biotechnological upcycling of plastic waste and other non-conventional feedstocks in a circular economy. Curr. Opin. Biotechnol. 2020, 62, 212–219. 10.1016/j.copbio.2019.11.011. 31881445

[ref9] Dissanayake L.; Jayakody L. N. Engineering Microbes to Bio-Upcycle Polyethylene Terephthalate. Frontiers in Bioengineering and Biotechnology 2021, 9, 656465 10.3389/fbioe.2021.656465. 34124018 PMC8193722

[ref10] Diao J.; Hu Y.; Tian Y.; Carr R.; Moon T. S. Upcycling of poly(ethylene terephthalate) to produce high-value bio-products. Cell Reports 2023, 42 (1), 111908 10.1016/j.celrep.2022.111908. 36640302

[ref11] Wei R.; Zimmermann W. Biocatalysis as a green route for recycling the recalcitrant plastic polyethylene terephthalate. Microbial Biotechnology 2017, 10 (6), 1302–1307. 10.1111/1751-7915.12714. 28401691 PMC5658586

[ref12] Rabot C.; Chen Y.; Lin S.-Y.; Miller B.; Chiang Y.-M.; Oakley C. E.; Oakley B. R.; Wang C. C. C.; Williams T. J. Polystyrene Upcycling into Fungal Natural Products and a Biocontrol Agent. J. Am. Chem. Soc. 2023, 145 (9), 5222–5230. 10.1021/jacs.2c12285. 36779837 PMC11062757

[ref13] Kim H. T.; Kim J. K.; Cha H. G.; Kang M. J.; Lee H. S.; Khang T. U.; Yun E. J.; Lee D.-H.; Song B. K.; Park S. J.; et al. Biological Valorization of Poly(ethylene terephthalate) Monomers for Upcycling Waste PET. ACS Sustainable Chem. Eng. 2019, 7 (24), 19396–19406. 10.1021/acssuschemeng.9b03908.

[ref14] Werner A. Z.; Clare R.; Mand T. D.; Pardo I.; Ramirez K. J.; Haugen S. J.; Bratti F.; Dexter G. N.; Elmore J. R.; Huenemann J. D.; et al. Tandem chemical deconstruction and biological upcycling of poly(ethylene terephthalate) to β-ketoadipic acid by Pseudomonas putida KT2440. Metabolic Engineering 2021, 67, 250–261. 10.1016/j.ymben.2021.07.005. 34265401

[ref15] Sullivan K. P.; Werner A. Z.; Ramirez K. J.; Ellis L. D.; Bussard J. R.; Black B. A.; Brandner D. G.; Bratti F.; Buss B. L.; Dong X.; et al. Mixed plastics waste valorization through tandem chemical oxidation and biological funneling. Science 2022, 378 (6616), 207–211. 10.1126/science.abo4626. 36227984

[ref16] Sadler J. C.; Wallace S. Microbial synthesis of vanillin from waste poly(ethylene terephthalate). Green Chem. 2021, 23 (13), 4665–4672. 10.1039/D1GC00931A. 34276250 PMC8256426

[ref17] Ravishankara A. R.; Daniel J. S.; Portmann R. W. Nitrous Oxide (N2O): The Dominant Ozone-Depleting Substance Emitted in the 21st Century. Science 2009, 326 (5949), 123–125. 10.1126/science.1176985. 19713491

[ref18] Rorrer N. A.; Notonier S. F.; Knott B. C.; Black B. A.; Singh A.; Nicholson S. R.; Kinchin C. P.; Schmidt G. P.; Carpenter A. C.; Ramirez K. J.; et al. Production of β-ketoadipic acid from glucose in Pseudomonas putida KT2440 for use in performance-advantaged nylons. Cell Reports Physical Science 2022, 3 (4), 100840 10.1016/j.xcrp.2022.100840.

[ref19] Suitor J. T.; Varzandeh S.; Wallace S. One-Pot Synthesis of Adipic Acid from Guaiacol in Escherichia coli. ACS Synth. Biol. 2020, 9 (9), 2472–2476. 10.1021/acssynbio.0c00254. 32786923

[ref20] Joo J. C.; Khusnutdinova A. N.; Flick R.; Kim T.; Bornscheuer U. T.; Yakunin A. F.; Mahadevan R. Alkene hydrogenation activity of enoate reductases for an environmentally benign biosynthesis of adipic acid. Chemical Science 2017, 8 (2), 1406–1413. 10.1039/C6SC02842J. 28616142 PMC5460604

[ref21] Pyne M. E.; Moo-Young M.; Chung D. A.; Chou C. P. Coupling the CRISPR/Cas9 System with Lambda Red Recombineering Enables Simplified Chromosomal Gene Replacement in Escherichia coli. Appl. Environ. Microbiol. 2015, 81 (15), 5103–5114. 10.1128/AEM.01248-15. 26002895 PMC4495200

[ref22] Sirasani G.; Tong L.; Balskus E. P. A Biocompatible Alkene Hydrogenation Merges Organic Synthesis with Microbial Metabolism. Angew. Chem., Int. Ed. 2014, 53 (30), 7785–7788. 10.1002/anie.201403148. PMC4127630 24916924

[ref23] Agapakis C. M.; Ducat D. C.; Boyle P. M.; Wintermute E. H.; Way J. C.; Silver P. A. Insulation of a synthetic hydrogen metabolism circuit in bacteria. Journal of Biological Engineering 2010, 4 (1), 3 10.1186/1754-1611-4-3. 20184755 PMC2847965

[ref24] Bibi Z.; Qader S. A. U.; Aman A. Calcium alginate matrix increases the stability and recycling capability of immobilized endo-β-1,4-xylanase from Geobacillus stearothermophilus KIBGE-IB29. Extremophiles 2015, 19 (4), 819–827. 10.1007/s00792-015-0757-y. 26001519

[ref25] Shakeri F.; Ariaeenejad S.; Ghollasi M.; Motamedi E. Synthesis of two novel bio-based hydrogels using sodium alginate and chitosan and their proficiency in physical immobilization of enzymes. Sci. Rep. 2022, 12 (1), 2072 10.1038/s41598-022-06013-0. 35136126 PMC8827098

[ref26] Raj K.; Partow S.; Correia K.; Khusnutdinova A. N.; Yakunin A. F.; Mahadevan R. Biocatalytic production of adipic acid from glucose using engineered Saccharomyces cerevisiae. In Metab Eng. Commun. 2018, 6, 28–32. 10.1016/j.meteno.2018.02.001. PMC5814376 29487800

[ref27] Gleizer S.; Ben-Nissan R.; Bar-On Y. M.; Antonovsky N.; Noor E.; Zohar Y.; Jona G.; Krieger E.; Shamshoum M.; Bar-Even A.; et al. Conversion of Escherichia coli to Generate All Biomass Carbon from CO2. Cell 2019, 179 (6), 1255–1263.e12. 10.1016/j.cell.2019.11.009. 31778652 PMC6904909

[ref28] Berrios-Rivera S. J.; Bennett G. N.; San K.-Y. The Effect of Increasing NADH Availability on the Redistribution of Metabolic Fluxes in Escherichia coli Chemostat Cultures. Metabolic Engineering 2002, 4 (3), 230–237. 10.1006/mben.2002.0228. 12616692

[ref29] Bai B.; Zhou J.-m.; Yang M.-h.; Liu Y.-l.; Xu X.-h.; Xing J.-m. Efficient production of succinic acid from macroalgae hydrolysate by metabolically engineered Escherichia coli. Bioresour. Technol. 2015, 185, 56–61. 10.1016/j.biortech.2015.02.081. 25747879

[ref30] Liang L.; Liu R.; Chen X.; Ren X.; Ma J.; Chen K.; Jiang M.; Wei P.; Ouyang P. Effects of overexpression of NAPRTase, NAMNAT, and NAD synthetase in the NAD(H) biosynthetic pathways on the NAD(H) pool, NADH/NAD+ ratio, and succinic acid production with different carbon sources by metabolically engineered Escherichia coli. Biochemical Engineering Journal 2013, 81, 90–96. 10.1016/j.bej.2013.09.018.

[ref31] Roosen M.; Mys N.; Kusenberg M.; Billen P.; Dumoulin A.; Dewulf J.; Van Geem K. M.; Ragaert K.; De Meester S. Detailed Analysis of the Composition of Selected Plastic Packaging Waste Products and Its Implications for Mechanical and Thermochemical Recycling. Environ. Sci. Technol. 2020, 54 (20), 13282–13293. 10.1021/acs.est.0c03371. 32985869

