# Supplementary material for: Microbial Upcycling of Waste PET to Adipic Acid
Source: ACS Cent Sci. 2023 Nov 1;9(11):2057–63. doi: 10.1021/acscentsci.3c00414 (PMC10683474; doi:10.1021/acscentsci.3c00414)
Supplement: Supplementary file 1 — oc3c00414_si_001.pdf [file oc3c00414_si_001.pdf]

Supplementary Information

Microbial upcycling of waste PET to adipic acid

Marcos Valenzuela-Ortega[1⊥], Jack T. Suitor[1⊥], Mirren F. M. White[1], Trevor Hinchcliffe[2] and Stephen Wallace[1*]

[1]Institute of Quantitative Biology, Biochemistry and Biotechnology, School of Biological Sciences, University of Edinburgh, Roger Land Building, Alexander Crum Brown Road, King's Buildings, Edinburgh, EH9 3FF, UK

[2]Impact Solutions Ltd, Impact Technology Centre, Fraser Road, Livingston, EH54 7BU, UK

**Email: stephen.wallace@ed.ac.uk*

Contents

S1 General Materials and Methods 3

S2 Plasmid Construction 5

S2.1 Plasmid maps 13

S3 Genome Modification 16

S4 Experimental Methods 19

S4.1 Protein expression in *E. coli* BL21(DE3) 19

S4.2 Whole cell biotransformation of terephthalic acid to adipic acid 19

S4.3 Fermentation reactions 19

S4.4 Bio-hydrogenation of *cis*,*cis*-muconic acid 19

S4.5 HPLC sample preparation from biotransformation reactions 20

S4.6 Cell immobilization in alginate hydrogels 21

S4.7 Terephthalic acid preparation from waste PET 22

S5 Supplementary data 23

S5.1 Protein expression 23

S5.2 Whole-cell mixing experiments 24

S5.3 Expression optimization and reaction optimization 26

S5.4 Biocompatible hydrogenation 28

S5.5 Cell immobilization experiments 28

S5.6 NMR Spectroscopy 33

S6 References 35

S1 General Materials and Methods

Unless otherwise stated, starting materials and reagents were obtained from commercial suppliers and were used without further purification. All water used experimentally was purified with a Suez Select purification system (18 MΩ.cm, 0.2 μM filter). The following analyte abbreviations are used throughout: adipic acid (AA), 2-hexenedioic acid (2HDA), *cis*,*cis*-muconic acid (ccMA), catechol (Cat), protocatechuic acid (PCA), terephthalic acid (TA), polyethylene terephthalate (PET). Disodium terephthalate was used for all experiments using TA, except when waste PET was used. 2HDA was synthesized as described previously[1]. Hot stamping foils were donated from API Foilmakers Ltd. in Livingston, UK. PET bottle samples were collected from domestic rubbish in Edinburgh, UK.

NMR: Proton nuclear magnetic resonance spectra (^{1}H NMR) were recorded using a Bruker AVA600 NMR spectrometer at the specified frequency at 298 K. Proton chemical shifts are expressed in parts per million (ppm, δ scale) and are referenced to residual protium in the NMR solvent. NMR solvents were used as purchased from commercial suppliers.

HPLC: High performance liquid chromatography (HPLC) analysis was carried out using a Thermo Fisher Scientific Dionex UltiMate 3000 Series UHPLC instrument equipped with a HyperSil Gold C18 column (150x3 mm x 3 μm). Analytes were detected at 206 nm and quantified by comparison to a caffeine internal standard, added to 50 μM final concentration. All HPLC solvents were purchased from commercial suppliers. Samples were analyzed using the following method:

Solvent A: Water+0.1% v/v trifluoroacetic acid (TFA).
Solvent B: Acetonitrile+0.1% v/v TFA

Time (min)	Flow rate (mL/min)	%B
0 to 10.2	0.4	5
10.2 to 20	0.4	15
20 to 20.2	0.4	50
20.2 to 35	0.4	5

Media Recipes and Microbiology

Lysogeny Broth (LB) Medium: Bacto-tryptone (10 g/L), yeast extract (5 g/L) and NaCl (10 g/L) were dissolved in Milli-Q H_2O. LB was autoclaved at 121 ˚C for 20 min, cooled and stored at room temperature. Solid media was made using the same recipe but with the addition of agar (15 g/L).

Terrific Broth (TB) Medium: Yeast extract (24 g/L), tryptone (20 g/L), glycerol (4 mL/L) and potassium phosphate buffer (72 mM K_2HPO_4; 12.5 g/L) were dissolved in in 1 L Milli-Q H_2O. TB was autoclaved at 121 ˚C for 20 min, cooled and stored at room temperature.

M9 Minimal Media (M9-Glucose, M9-Glycerol and M9-CA) stock solutions: A 5X stock solution of M9 salts containing Na_2HPO_4, (30 g/L), KH_2PO_4 (15 g/L), NH_4Cl (5 g/L) and NaCl (2.5 g/L) was prepared and autoclaved at 121 ˚C for 20 min, cooled and stored at room temperature. A stock solution of 5X M9CA salts was prepared with the further addition of casamino acids (25 g/L).

Stock solutions of 20% w/v glucose and 20% w/v glycerol were prepared and autoclaved at 121 ˚C for 20 min, cooled and stored at room temperature. Stock solutions of $MgSO_4$ (1 M), $CaCl_2$ (50 mM), thiamine hydrochloride (10 mg/mL) were prepared and filter sterilized.

M9-Glucose: For 1 L media: 5X M9 salts (200 mL, 1X final concentration), $MgSO_4$ (2 mL, 2 mM final concentration), $CaCl_2$ (2 mL, 0.1 mM final concentration), and glucose (25 mL, 0.5% w/v final concentration) were combined and sterile water was added to a final vol of 1 L.

M9-Glycerol: For 1 L media: 5X M9 salts (200 mL, 1X final concentration), $MgSO_4$ (2 mL, 2 mM final concentration), $CaCl_2$ (2 mL, 0.1 mM final concentration), and glycerol (25 mL, 0.5% w/v final concentration) were combined and sterile water was added to a final vol of 1 L.

M9-CA: For 1 L media: 5X M9CA salts (200 mL, 1X final concentration), $MgSO_4$ (2 mL, 2 mM final concentration), $CaCl_2$ (2 mL, 0.1 mM final concentration), thiamine hydrochloride (0.1 mL, 1 μg/mL final concentration), glucose (25 mL, 0.5% w/v final concentration) were combined and sterile water was added to a final vol of 1 L.

Unless stated otherwise, *E. coli* cells were cultured at 37 ˚C with shaking at 220 rpm in an incubator shaker with a 5.1 cm orbit throw. Optical densities of *E. coli* cultures were determined using a DeNovix DS-11 UV/Vis spectrophotometer by measuring absorbance at 600 nm. For bacterial growth and protein induction, antibiotics required by each strain were added to media at the following concentrations: 100 μg/mL ampicillin, 30 μg/mL chloramphenicol, 50 μg/mL kanamycin, 25 μg/mL spectinomycin.

Molecular Biology

All synthetic genes were codon-optimized for *E. coli* BL21(DE3) and synthesized using GeneArt™ (Thermo Scientific). Oligonucleotide primers were synthesized by Integrated DNA Technologies. OneTaq 2X (New England Biolabs, NEB) was used for colony PCRs, Phusion High-Fidelity DNA Polymerase (NEB) was used for all other PCR reactions, following manufacturer recommendations.

Products from PCR were gel purified with a Zymoclean Gel DNA Recovery Kit (Zymo Research). All restriction enzymes and T4 DNA ligase were purchased from Thermo Fisher and used following manufacturer recommendations. Plasmid DNA was purified with a Miniprep Kit (Qiagen) from *E. coli* DH5α. All generated plasmids were confirmed by colony PCR and Sanger sequencing (Azenta).

Construction of all recombinant plasmid DNA was done in *E. coli* DH5α. All chemically competent cells were prepared via treatment with calcium chloride[2] and transformed via heat-shock at 42 ˚C for 45 sec, followed by a recovery step in 1 mL of SOC media for 1 h at 37 ˚C and then plating on LB agar containing appropriate antibiotics and overnight incubation at 37 ˚C.

For SDS-PAGE, 12-well 12% acrylamide Bis-Tris NuPAGE gels and 4-12% Bis-TRIS NuPAGE Bolt gels (Thermo Scientific) containing an unstained Precision plus standard ladder (BioRad) were used to analyze samples. Gels were run in 1X MES buffer (Novagen) at 50 V for 30 min followed by 150 V for 2 h.

Homology-mediated DNA assembly was done with SLiCE (Seamless Ligation Cloning Extract)[3] Ligation Independent Cloning (LIC)[4], or AquaCloning[5]. A typical SLiCE assembly was prepared as follows: a 10 µL reaction was set containing 1 µL of SLiCE extract (prepared from *E. coli* JM109 according to the published protocol[6]), linearised vector (50 ng) and insert(s) at a molar concentration ration of 3:1 (insert:vector). SLiCE cloning reactions were incubated at 37 °C for 1 h before transformation of *E. coli* DH5α. Linearized DNA used for assemblies was gel-extracted.

Modular cloning was done following using JUMP (Joint Universal Modular Plasmids) backbones and plasmids and following the protocols indicated in [7].

S2 Plasmid Construction

Table S1. List of plasmids used in this study.

Plasmid name	Description	Reference
pAA	pETDuet backbone (pBR322/Rop oriV, AmpR, *lacI*, T7 IPTG induction), monocistronic *catA, bcER.*	1
pQLinkN-*bcER-catA* (pAA2)	pQLinkN backbone (pUC oriV, AmpR, *lacI*, IPTG induction), monocistronic *catA, bcER.*	1
pAA'	pQLinkN backbone (pUC oriV, AmpR, *lacI*, IPTG induction), monocistronic *gcoA, gcoB, catA, bcER.*	1

pVan1	pBR322/Rop oriV, AmpR, T7 IPTG induction, and polycistronic cassette (coding *tphA1*, *tphA2*, *tphB2*, *dcddh*).	8
pQLinkN-*aroY-kpdB*	pQLinkN backbone (pUC oriV, AmpR, *lacI*, IPTG induction), monocistronic *aroY, kpdB.*	This work
pAA3	pQLinkN backbone (pUC oriV, AmpR, *lacI*, IPTG induction), monocistronic *aroY, kpdB, bcER, catA*	This work
pPCA1	pGro7-derived backbone (p15a oriV, CamR, T7 IPTG induction) and polycistronic cassette from pVan1 coding *tphA1*, *tphA2*, *tphB2*, *dcddh*.	This work
pAA4	pET22b(+) backbone (pBR322/Rop oriV, AmpR, *lacI*, T7 IPTG induction), cassette coding: *aroY*, *kpdB*, *bcER*, *catA*	This work
pPCA2	pET22b(+) backbone (pBR322/Rop oriV, AmpR, *lacI*, T7 IPTG induction), polycistronic cassette (coding *tphA1*, *tphA2*, *tphB2*, *dcddh*)	This work
pAA5	pGro7 backbone (p15a oriV, CamR, T7 IPTG induction), cassette coding: *aroY*, *kpdB*, *bcER*, *catA*	This work
pGro7	Chaperone expression	Takara Bio Inc.
pX2-Cas9	Cas9 expression for CRISPR-mediated genome integration. (Addgene plasmid # 85811)	pX2-Cas9 was a gift from Ryan Gill
pSS9	Backbone for construction of donor DNA for CRISPR-mediated genome integration. (Addgene plasmid #71655)	9
SS9-gRNA	gRNA expression for CRISPR-mediated genome integration. (Addgene #71656)	9
pIS6_PCA	pSS9 modified to integrate PCA cassette in IS6 locus.	This work
pSS3_PCA	pSS9 modified to integrate PCA cassette in SS3 locus.	This work
pSS9_PCA	pSS9 modified to integrate PCA cassette in SS9 locus.	This work
pIS6_gRNA	SS9-gRNA modified to generate a gRNA targeting IS6 locus.	This work
pSS3_gRNA	SS9-gRNA modified to generate a gRNA targeting SS3 locus.	This work
pSS9_gRNA	SS9-gRNA modified to generate an alternative gRNA targeting SS3 locus.	This work
pPCAX-1	pPCA1 with *fdh* expressed by constitutive J23107 promoter.	This work
pPCAX-2	pPCA1 with *fdh* expressed by constitutive J23100 promoter.	This work
pPCAX-3	pPCA1 with *fdh* expressed by constitutive J23119 promoter.	This work

pQLinkN-AroY-KpdB and pAA3

The plasmid pAA3, combining monocistronic genes *aroY*, *kpdB*, *catA* and *bcER*, was constructed using a pQLinkN backbone[10]. The aroY and kpdB synthetized genes (sequences given in Table S2) were amplified via PCR (primers described in Table S3) and initially cloned into the pQLinkN vector using BamHI and NotI enzymes. pQLinkN-*aroY*-*kpdB* was generated by digesting pQLinkN-*aroY* pQLinkN-*kpdB* with PacI and SwaI, respectively, and assembling them via SLiCE cloning. Plasmid pAA3 was constructed by digesting pQLinkN-*bcER*-*catA*[1] and pQLinkN-*aroY*-*kpdB* with PacI and SwaI, respectively, and assembling them via SLiCE cloning.

pAA4

Sequences coding for AroY, KpdB, CatA, and BcER, and the pET22b(+) vector were amplified with primers in Table S3. These oligos introduced the RBS sequences BBa_B0035, BBa_B0032, BBa_B0030 and BBa_B0064, upstream of *bcER*, *aroY*, *kpdB*, and *catA* genes, respectively. The resulting fragments were assembled using SLiCE cloning.

pPCA1

The backbone of pGro7 (Takara Bio Inc.) and the expression cassette of the pVan1 plasmid[8] (coding for *tphA1*, *tphA2*, *tphB2*, *dcddh*) were PCR amplified (using primers in Table S3) and combined using SLiCE cloning.

pPCA2 and pAA5

The expression cassettes and backbones of the pAA4 and pPCA1 plasmids were exchanged to generate pPCA2 and pAA5. Insert and backbones were amplified via PCR using the primers shown in Table S3, and the purified PCR products were assembled via SLiCE cloning. These PCR reactions used Phusion polymerase High GC buffer instead of standard 5x Phusion HF buffer.

pPCAX plasmids

Sequence coding for Fdh (Table S2) was synthesised flanked with BsaI restriction sites AATG and GCTT and was assembled into vector pJUMP27-1A with RBS B0034, terminator L3S1P51 and promoter J23107 (weak), J23100 (medium), or J23119 (strong). Genes encoding Fdh were amplified with primers shown in Table S3.

Table S2. Nucleotide sequences of protein coding sequences used in this study

Protein coded	Nucleotide sequence (5' to 3')
BcER	ATGGGAAAATACAAGAAACTGTTCGAAACCGTGAAAATCCGTAACGTGG<br>AACTGAAAAATCGTTATGCAATGGCACCGATGGGTCCGCTGGGTTTAGCA<br>GATGCAGAAGGTGGTTTTAATCAGCGTGGTATTGAGTATTATACCGCACG<br>TGCCCGTGGTGGCACCGCACTGATTATTACCGGTGTTACCTTTGTTGATAA<br>CGAGGTTGAAGAACATGGTATGCCGAATGTTCCGTGTCCGACACATAATC<br>CGGTTCATTTTGTTCGTACCAGCAAAGAAATGACCGAACGTATTCATGCA<br>TACGATAGCAAAATCTTTCTGCAGATGAGCGCAGGTTTTGGTCGTGTTAC<br>CATTCCGACCAATCTGGGTGAATATCCGCCTGTTGCACCGAGTCCGATTCC<br>GCATCGTTGGCTGGATAAAACCTGTCGTGAACTGACCGTTGAAGAAATTC<br>ATAGCATTGTGCGCAAATTTGGTGATGGTGCATTTAATGCAAAACGCGCA<br>GGCTTTGATGGTGTTCAGATTCATGCAGTTCATGAAGGTTATCTGCTGGAT<br>CAGTTTGCAATCGCCTTTTTTAACAAACGTACCGATGCCTATGGTGGACCG<br>CTGGAAAATCGTCTGCGTTTTGCCCGTGAAATTGTGGAAGAAATTAAACA<br>GCGTTGCGGTGAAGATTTTCCGGTTACACTGCGTTTTAGTCCGAAAAGCTT<br>TATCAAAGATTGGCGTGAAGGTGCACTGCCTGGTGAAGAATTTGAAGAAA<br>AAGGTCGTGATCTGGATGAAGGTATTGAAGCAGCAAAACTGCTGGTTAGC<br>TATGGTTATGATGCACTGGATGTTGATGTTGGCAGCTATGATAGTTGGTG<br>GTGGTCACATCCGCCTATGTATCAGAAAAAAGGTCTGTATATTCCGTATG<br>CGCGTCTGGTTAAAGAAGCAGTTGACGTTCCGGTTCTGTGTGCAGGTCGT<br>ATGGATAATCCGGATCTGGCACTGGCAGCACTGGAAGATGGTGCCTGTGA<br>TATTATCAGCCTGGGTCGTCCGCTGCTGGCCGATCCTGATTATGTTAATAA<br>ACTGCGTATTGGTCAGGTGGCAGATATTCGTCCGTGTCTGAGCTGTCATG<br>AAGGCTGTATGGGTCGTATTCAAGAATATTCAAGCCTGGGTTGTGCAGTT<br>AATCCGGCAGCATGTCGTGAAAAAGAAGCCGCACTGACACCGGCACTGA<br>AAAAAAAGCGTGTTCTGATTGCCGGTGGTGGTGTTGCAGGTTGTGAAGCA<br>GCCCGTGTTCTGGCCCTGCGTGGTCATGAACCGGTGATTTTTGAAAAAAG<br>CAATCGCTTAGGTGGCAATCTGATTCCTGGTGGCGCACCGGATTTTAAAG<br>AAGATGATCTGGCGCTGGTTGCATGGTATGAACATACCCTGGAACGTCTG<br>GGTGTTGAAATTCATCTGAATACAGCACTGACCAAAGAAGAAATCCTGGC<br>AGCAAATGTTGATGCAGTGCTGATTGCAACCGGTAGTAATCCGAAAATTC<br>TGCCGCTGGATGGTAAAAACAAAGTGTTTACCGCAGAAGATGTTCTGCTG<br>GACAAAGTTGATGCCGGTCAGCATGTTGTTATTGTTGGTGGCGGTCTGGTT<br>GGTTGTGAACTGGCCCTGAATCTGGCCGAAAAAGGTAAAGATGTTAGCCT<br>GGTTGAAATGCAGGATAAACTGTTAGCAGTTAATGGTCCGCTGTGTCATG<br>CAAATAGCGATATGCTGGAACGCCTGGTTCCGTTTAAAGGTGTTCAGGTT<br>TATACCTCCAGCAAAATTGTTGATACCACCGAAAAAACCGCAGTTGTTGA<br>TGTGGATGGTGAACTGCGCGAAATTGAAGCCGATAGTATTGTTCTGGCAG<br>TTGGTTATAGCGCAGAGAAAAGCCTGTATGAAGATCTGAAATTTGAAGTG<br>GCCGATCTGCATGTTGTGGGTGATGCACGTAAAGTTGCCAATATTATGTA<br>TGCAATCTGGGATGCCTATGAAGTGGCAGCCAATTTAGCTTGATGA
CatA	ATGACCGTGAAAATTAGCCATACCGCAGATATTCAGGCCTTTTTAACCG<br>TGTTGCAGGTCTGGATCATGCAGAAGGTAATCCGCGTTTTAAGCAGATTA<br>TTCTGCGTGTTCTGCAGGATACCGCACGTCTGATTGAAGATCTGGAAATT<br>ACCGAAGATGAATTTTGGCATGCCGTGGATTATCTGAATCGTTTAGGTGG<br>TCGTAATGAAGCAGGTCTGCTGGCAGCCGGTCTGGGTATTGAACATTTTC<br>TGGATCTGCTGCAGGATGCAAAAGATGCCGAAGCAGGTTTAGGCGGTGGT<br>ACACCGCGTACCATTGAAGGTCCGCTGTATGTTGCGGGTGCACCGCTGGC<br>ACAGGGTGAAGCACGTATGGATGATGGCACCGATCCGGGTGTTGTTATGT<br>TTCTGCAGGGTCAAGTTTTTGATGCAGATGGTAAACCTCTGGCAGGCGCA<br>ACCGTTGATCTGTGGCATGCAAATACCCAGGGCACCTATAGCTATTTTGA

	TAGCACCCAGAGCGAATTTAATCTGCGTCGTCGTATTATTACCGATGCGG<br>AAGGTCGTTATCGTGCACGTAGCATTGTTCCGAGCGGTTATGGTTGTGATC<br>CGCAGGGTCCGACACAAGAATGTCTGGACCTGCTGGGTCGTCATGGTCAG<br>CGTCCGGCACATGTTCATTTTTTCATTAGCGCACCGGGTCATCGTCATCTG<br>ACCACACAGATTAACTTTGCCGGTGATAAATATCTGTGGGATGATTTTGC<br>CTATGCAACCCGTGATGGTCTGATTGGTGAACTGCGTTTTGTTGAAGATGC<br>AGCAGCAGCACGTGATCGTGGTGTTCAGGGTGAACGTTTTGCAGAACTGA<br>GCTTTGATTTTCGCCTGCAGGGTGCCAAAAGTCCGGATGCAGAAGCCCGT<br>AGCCATCGTCCGCGTGCACTGCAAGAAGGGAATTCATGATGA
AroY	ATGACTGCGCCAATTCAGGATCTGCGCGACGCGATCGCGTTGTTACAGCA<br>GCATGACAACCAGTATTTAGAGACCGATCACCCAGTTGACCCGAACGCGG<br>AACTGGCAGGGGTATATCGTCATATTGGCGCGGGAGGCACGGTGAAACG<br>GCCGACCCGTATTGGACCGGCAATGATGTTTAACAATATTAAGGGGTACC<br>CGCATTCACGTATTCTTGTGGGCATGCACGCCTCTCGGCAACGTGCGGCA<br>CTGCTGCTGGGCTGCGAGGCATCGCAGTTGGCTTTAGAAGTGGGGAAGGC<br>CGTAAAGAAACCGGTCGCACCAGTTGTGGTCCCGGCAAGTTCCGCACCTT<br>GTCAGGAACAGATCTTTCTTGCAGATGACCCGGACTTCGACCTGCGCACC<br>TTGCTTCCCGCCCCGACGAATACGCCGATTGACGCGGGTCCGTTTTTTGT<br>CTGGGACTCGCTTTAGCGTCCGATCCTGTCGATGCCTCCCTGACGGATGTG<br>ACGATTCACCGCCTGTGCGTGCAGGGCCGTGATGAATTAAGCATGTTTTT<br>GGCGGCGGGGCGTCACATCGAAGTCTTCCGCCAGAAGGCTGAAGCCGCG<br>GGTAAGCCCCTTCCGATCACGATTAACATGGGCTTAGATCCAGCTATTTAT<br>ATCGGGGCATGTTTTGAAGCGCCAACCACTCCCTTTGGCTACAACGAACT<br>GGGGGTGGCGGGTGCCCTGCGGCAACGCCCCGTGGAACTGGTCCAAGGA<br>GTTTCTGTACCTGAAAAAGCGATTGCGCGCGCAGAAATCGTCATTGAAGG<br>AGAATTGTTGCCAGGGGTACGTGTCCGCGAAGATCAGCACACGAATTCAG<br>GTCATGCGATGCCTGAGTTTCCAGGCTACTGCGGTGGGGCAAATCCTTCT<br>CTGCCCGTTATCAAAGTTAAGGCGGTGACTATGCGTAACAACGCCATCCT<br>GCAAACGCTTGTGGGACCGGGTGAAGAACATACGACCTTGGCCGGTCTGC<br>CGACCGAAGCATCTATTTGGAACGCGGTGGAAGCCGCGATCCCCGGTTTC<br>CTCCAAAACGTTTATGCCCACACGGCAGGAGGGGGGAAATTCTTAGGTAT<br>CCTGCAGGTTAAAAAGCGTCAGCCGGCTGACGAGGGTCGTCAAGGACAA<br>GCCGCATTGCTGGCATTAGCGACCTACTCCGAGCTGAAAAACATCATCCT<br>GGTTGATGAAGATGTCGACATTTTTGATTCTGACGATATCCTGTGGGCCAT<br>GACAACCCGGATGCAGGGTGACGTTAGCATCACCACGATCCCCGGCATTC<br>GCGGTCATCAGTTAGACCCGAGCCAAACTCCTGAGTATTCACCCAGTATC<br>CGCGGTAACGGTATTAGCTGCAAGACCATCTTTGACTGCACCGTGCCGTG<br>GGCCCTTAAAAGTCATTTTGAGCGTGCGCCATTCGCTGACGTTGACCCGC<br>GCCCTTTTGCCCCGGAATATTTCGCCCGTCTCGAAAAAAACCAAGGCAGC<br>GCGAAATCATCA
KpdB	ATGAAACTCATTATCGGCATGACCGGCGCGACCGGCGCACCTTTAGGCGT<br>TGCCTTGTTGCAAGCTCTGCGCGATATGCCGGAGGTTGAAACTCACCTGG<br>TAATGTCGAAATGGGCTAAAACCACGATCGAGCTGGAAACTCCTTGGACG<br>GCGCGTGAAGTAGCAGCCTTGGCAGATTTTAGCCATTCACCGGCTGACCA<br>GGCTGCGACGATCAGTTCAGGTAGCTTTCGCACTGATGGGATGATTGTTA<br>TTCCCTGTTCAATGAAAACCCTTGCGGGTATCCGTGCCGGCTATGCCGAA<br>GGCTTGGTCGGACGCGCAGCGGACGTTGTTCTGAAAGAGGGGCGTAAACT<br>GGTCCTGGTTCCGCGGGAAATGCCGCTGTCGACAATTCATCTGGAAAACA<br>TGCTGGCTCTGTCCCGGATGGGTGTGGCAATGGTTCCCCCCATGCCGGCG<br>TATTACAATCACCCGGAGACCGTTGACGACATTACCAATCACATCGTTAC<br>CCGTGTATTAGATCAGTTTGGCCTGGACTATCACAAAGCGCGCCGCTGGA<br>ACGGCCTTCGTACAGCCGAACAGTTTGCCCAAGAAATTGAATCATCA
TphA1	ATGAATCATCAGATACATATTCACGATTCGGATATAGCATTTCCTTGCGCA<br>CCCGGGCAATCTGTTTTGGATGCGGCTCTGCAAGCAGGCATAGAATTGCC

	ATACAGCTGCCGTAAAGGTTCCTGTGGCAACTGCGCATCAGCCCTATTGG<br>ATGGGAATATTACGTCTTTTAATGGCATGGCTGTCCGATCGGAGCTGTGC<br>ACATCAGAGCAAGTACTCTTGTGTGGCTGCACTGCTGCATCAGATATCAG<br>AATTCAACCGAGCAGCTTTCGGAGGCTGGACCCGGAAGCTCGCAAGAGG<br>TTTACTGCAAAGGTGTACTCCAATACTCTGGCGGCACCGGATGTGTCTCTC<br>CTCCGTCTCAGGTTACCAGTTGGCAAGCGAGCGAAATTCGAAGCGGGCCA<br>ATATTTACTTATTCACCTTGATGATGGCGAATCACGGTCATACAGTATGGC<br>CAATCCGCCCCACGAGAGCGATGGGATAACGCTTCACGTACGCCACGTTC<br>CTGGCGGACGATTCTCCACAATAGTGCAACAACTGAAAAGCGGCGATAC<br>ATTGGAAATTGAGCTGCCCTTTGGCTCGATTGCGCTCAAACCGGATGATA<br>CGAGGCCACTGATATGCGTTGCTGGGGGTACGGGTTTTGCGCCAATTAAA<br>TCAGTTCTCGACGATTTAGCTAAGCGAAAAGTTCAGCGCGACATCACCTT<br>GATCTGGGGCGCCAGGAATCCTAGCGGCTTATACTTGCCTAGCGCGATCG<br>ACAAGTGGAGGAAAACATGGCCACAGTTCCGATACATTGCGGCAATCAC<br>AGACTTGGGCAACGTTCCGGCAGACGCGCACGCTGGACGTGTTGACGATG<br>CCTTGCGCACCCACTTTGGAAACCTTCACGATCATGTTGTTCATTGCTGCG<br>GGTCGCCTAGCCTGGTTCAATCGGTCCGGACCGCTGCCAGCGACATGGGA<br>CTTTTGGCCCAGAATTTTCACGCAGACGTGTTTGCAACCAGCCCGACGGG<br>TTCCCATTAA
TphA2	ATGCAAGAGTCAATTATCCAGTGGCATGGTGCAACAAACACTCGGGTACC<br>ATTTGGCATCTATACTGACACTGCCAATGCAGATCAAGAGCAACAGAGGA<br>TCTATCGGGGGGAGGTCTGGAACTATTTGTGTCTGGAGTCAGAAATCCCC<br>GGAGCGGGAGATTTCCGAACAACCTTCGCGGGAGAAACGCCAATTGTAG<br>TTGTTCGCGACGCTGATCAAGAAATCTACGCCTTTGAAAACCGGTGTGCC<br>CACCGCGGAGCACTGATCGCGTTGGAGAAGAGTGGAAGGACGGACTCTT<br>TCCAATGCGTGTATCATGCCTGGTCGTACAACCGTCAAGGCGATCTGACC<br>GGCGTTGCGTTTGAAAAAGGGGTCAAGGGCCAAGGTGGTATGCCGGCCTC<br>CTTTTGCAAGGAAGAGCACGGGCCGCGAAAGCTCAGGGTCGCTGTGTTCT<br>GCGGACTTGTCTTTGGTTCATTTAGCGAGGACGTACCCTCGATCGAGGATT<br>ATCTGGGTCCTGAAATCTGCGAACGCATAGAGCGCGTTCTGCACAAACCC<br>GTAGAGGTCATAGGCCGGTTCACGCAGAAGCTGCCTAACAATTGGAAACT<br>TTACTTTGAGAACGTAAAAGATTCCTACCACGCATCTCTCCTACATATGTT<br>TTTTACGACATTTGAGCTCAACCGGTTATCACAAAAGGGGGGAGTAATTG<br>TGGATGAGTCAGGCGGGCATCATGTATCATACTCTATGATCGATCGCGGT<br>GCCAAAGACGATTCTTACAAAGATCAAGCGATCAGGAGCGATAACGAAC<br>GGTACCGGCTCAAAGACCCTTCATTACTCGAAGGTTTCGAAGAATTCGAG<br>GACGGCGTAACTCTTCAGATCTTGTCCGTTTTCCCAGGCTTTGTACTTCAA<br>CAGATTCAGAACAGCATAGCAGTTAGACAACTGCTGCCTAAATCGATCAG<br>TAGTAGCGAATTGAACTGGACATACCTAGGCTATGCTGACGATTCGGCTG<br>AGCAGAGAAAAGTCCGGCTGAAACAGGCCAACCTCATAGGTCCAGCAGG<br>GTTTATCTCTATGGAAGATGGTGCAGTTGGCGGCTTCGTTCAGCGCGGGA<br>TTGCTGGGGCCGCCAATCTCGATGCCGTTATCGAGATGGGCGGTGACCAC<br>GAAGGCTCCTCAGAGGGACGAGCGACAGAAACAAGCGTAAGAGGGTTTT<br>GGAAAGCCTATCGTAAACATATGGGCCAAGAAATGCAAGCATAA
TphB2	ATGATAAACGAAATACAGATAGCCGCCTTTAATGCGGCCTATGCGAAAAC<br>GATTGATAGTGATGCCATGGAACAGTGGCCCACGTTCTTCACCAAGGATT<br>GCCATTACTGCGTGACGAACGTGGATAACCACGATGAGGGCCTGGCTGCC<br>GGAATCGTGTGGGCTGACTCACAAGATATGTTGACTGACAGAATTTCCGC<br>ATTACGCGAAGCAAACATTTACGAGCGGCATCGTTATCGGCATATCCTCG<br>GATTACCATCGATTCAATCTGGAGACGCGACGCAAGCGTCCGCGTCTACT<br>CCGTTCATGGTCCTTCGAATTATGCACACGGGTGAGACAGAAGTCTTTGC<br>CAGCGGGGAATACTTAGACAAATTTACCACAATTGATGGCAAGCTCCGCT<br>TACAAGAACGAATTGCGGTCTGTGATTCAACGGTCACCGACACTCTGATG<br>GCACTGCCTCTGTAA

DCDDH	ATGACAATTGTGCACCGGCGCTTAGCACTCGCGATCGGTGATCCACATGG CATAGGCCCTGAAATCGCCTTGAAGGCTCTGCGACAACTCAGCGCGAATG AGCGATCCTTAATCAAAGTGTATGGTCCATGGAGCGCACTTGAACAGGCC GCTCAAATTTGCCAGATGGAATCTCTCCTCCAGGATCTGATACACGAAGA AGCTGGATCATTAGCGCAACCCGCGCAGTGGGGAGAAATCACCCCCCAG GCGGGACTTAGCACTGTCCAGAGTGCGACTGCTGCCATTCGGGCGTGCGA GAATGGAGAAGTGGATGCTGTCATCGCATGCCCGCACCACGAAACTGCG ATCCATCGCGCTGGTATAGCATTCAGCGGCTACCCGTCTCTGCTTGCCAAC GTTCTTGGTATGAACGAAGATCAGGTCTTTCTTATGCTTGTAGGGGCCGGC TTGCGCATCGTACACGTGACCCTCCATGAATCAGTAAGGAGCGCCTTGGA ACGGTTATCGCCGCAGCTGGTTGTAAATGCAGTTCAGGCAGCAGTGCAGA CGTGCACACTATTGGGGGTTCCCAAACCACAAGTGGCCGTGTTCGGTATA AACCCACATGCGTCCGAAGGGCAACTGTTTGGGCTGGAAGACAGTCAAAT CACCGCGCCGGCCGTTGAAACATTACGTAAGTGCGGACTCGCGGTCGACG GTCCTATGGGAGCCGACATGGTTTTGGCCCAACGCAAACATGATCTCTAT GTGGCTATGCTGCATGATCAAGGACACATACCGATTAAACTTTTGGCGCC GAATGGGGCCTCAGCTCTGTCTATAGGTGGGCGTGTGGTCTTGAGTTCCG TAGGCCACGGAAGCGCTATGGACATAGCAGGTCGTGGTGTCGCCGATTCA ACCGCGCTGTTGAGGACTATAGCCCTATTGGGTGCGCAGCCCGGTTAA
FDH	ATGGCTAAAGTTCTGTGCGTTCTGTACGACGACCCGGTTGACGGTTACCC GAAAACCTACGCTCGTGACGACCTGCCGAAAATCGACCACTACCCGGGTG GTCAGACCCTGCCGACCCCGAAAGCTATCGACTTCACCCCGGGTCAGCTG CTGGGTTCTGTTTCTGGTGAACTGGGTCTGCGTAAATACCTGGAATCTAAC GGTCACACCCTGGTTGTTACCTCTGACAAAGACGGTCTGGACTCTGTTTTC GAACGTGAACTGGTTGACGCTGACGTTGTTATCTCTCAGCCGTTCTGGCCG GCTTACCTGACCCCGGAACGTATCGCTAAAGCTAAAAACCTGAAACTGGC TCTGACCGCTGGTATCGGTTCTGACCACGTTGACCTGCAATCTGCTATCGA CCGTAACGTTACCGTTGCTGAAGTTACCTACTGCAACTCTATCTCTGTTGC TGAACACGTTGTTATGATGATCCTGTCTCTGGTTCGTAACTACCTGCCGTC ACACGAATGGGCTCGTAAAGGTGGTTGGAACATAGCTGACTGCGTAAGCC ACGCTTACGACCTGGAAGCTATGCACGTTGGTACCGTTGCTGCTGGTCGT ATCGGTCTGGCTGTTCTGCGTCGTCTGGCTCCGTTCGACGTTCATCTGCAC TACACCGACCGTCACCGTCTGCCGGAATCTGTTGAAAAAGAACTGAACCT GACCTGGCACGCTACCCGTGAAGATATGTACCCGGTTTGCGACGTTGTTA CCCTGAACTGCCCGCTGCACCCGGAAACCGAACACATGATCAACGACGA AACCCTGAAACTGTTCAAACGTGGTGCTTACATCGTTAACACCGCTCGTG GTAAACTGTGCGACCGTGACGCTGTTGCTCGTGCTCTGGAATCTGGTCGTC TGGCTGGTTATGCGGGTGACGTGTGGTTCCCCCAGCCGGCTCCGAAAGAC CACCCGTGGCGTACCATGCCGTACAACGGTATGACCCCGCACATCTCTGG TACCACCCTGACCGCTCAGGCTCGTTACGCTGCTGGTACCCGTGAAATCCT GGAATGCTTCTTCGAAGGTCGTCCGATCCGTGACGAATACCTGATCGTTC AGGGTGGTGCTCTGGCTGGTACCGGTGCTCACTCTTACTCTAAAGGTAAC GCTACCGGTGGTTCTGAAGAAGCTGCTAAATTCAAAAAAGCTGTTTAA

Table S3. Oligonucleotide primers used for plasmid construction.

Plasmid	Template	Sense	Sequence
pQLinkN-*aroY-kpdB*	*aroY*	F	CAGAGCTCAGGATCCATGACTGCGCCAAT TCAGGA
	aroY	R	CTGACGATGCGGCCGCTCATCATTTCGCG CTGCCTTGGTTTT
	kpdB	F	CAGAGCTCAGGATCCATGAAACTCATTAT CGGCATGA
	kpdB	R	CTGACGATGCGGCCGCTCATCATTCAATT TCTTGGGCAAACTG
pAA4	*bcER*	F	TTGTTTAACTTTAAGATTAAAGAGGAGAAAATAAAA TGGGAAAATACAAGAAACTGTTCG
	bcER	R	CTTTCCTGTGTGACTCTAGATCATCACGCGGCTGCTA A
	catA	F	AGTTTGCCCAAGAAATTGAAGTTACCAAAGAGGGGA AAATGACCGTGAAAATTAGCCA
	catA	R	TTGTTAGCAGCCGGATCTCACTCATCATGAATTCCCT TCTTG
	aroY	F	CAGCCGCGTGATGATCTAGAGTCACACAGGAAAGAA TTAAATGACTGCGCCAATTCAGGA
	aroY	R	TCTCCTCTTTAATTATTCGTTCATCATTTCGCGCTGCC TTGGTTT
	kpdB	F	AGCGCGAAAACGAATAATTAAAGAGGAGAAATAAT AAATGAAACTCATTATCGGCATGAC
	kpdB	R	TTTCCCCTCTTTGGTAACTCATCATTCAATTTCTTGGG CAAACT
	pET22b(+)	F	AGAAGGGAATTCATGATGAGTGAGATCCGGCTGCTA AC
	pET22b(+)	R	TTTATTTTCTCCTCTTTAATCTTAAAGTTAAACAAAAT TATTTCTAGAGG
pPCA1	pGro7	F	GGGTCTTGAGGGGTTTTTTGttcgaatttctgccattcatcc
	pGro7	R	CCCTATAGTGAGTCGTATTAaagcttatcgatgataagctgtc
	pVan1 cassette	F	gacagcttatcatcgataagcttTAATACGACTCACTATAGGGGAA
	pVan1 cassette	R	ggatgaatggcagaaattcgaaCAAAAAACCCCTCAAGACC
pPCA2	pPCA1 cassette	F	TAATTTTGTTTAACTTTAAGAAGG AGATATACCATGATGAATCATCA GATACA
	pPCA1 cassette	R	TTGTTAGCAGCCGGATCTCATTA ACCGGGCTGCGCACC
	pAA4 vector	F	TGGGTGCGCAGCCCGGTTAATGA GATCCGGCTGCTAAC
	pAA4 vector	R	TCATCATGGTATATCTCCTTCTTA AAGTTAAACAAAATTATTTCTAG AGG
pAA5	pGro7 backbone	F	TGCAAGAAGGGAATTCATGAttcga atttctgccattcatcc
	pGro7 backbone	R	TTTATTTTCTCCTCTTTAATaagcttat cgatgataagctgtc
	pAA4 cassette	F	agcttatcatcgataagcttATTAAAGAGGA GAAAATAAAATGG
	pAA4 cassette	R	atgaatggcagaaattcgaaTCATGAATTCC CTTCTTG
pPCAX	pJUMP27-1A:Fdh	F	tttgacagcttatcatcgatAAGCTcttctagagcgtctctggag
		R	CCTATAGTGAGTCGTATTAAAGCTgtacgtctcacattagcgaac

S2.1 Plasmid maps

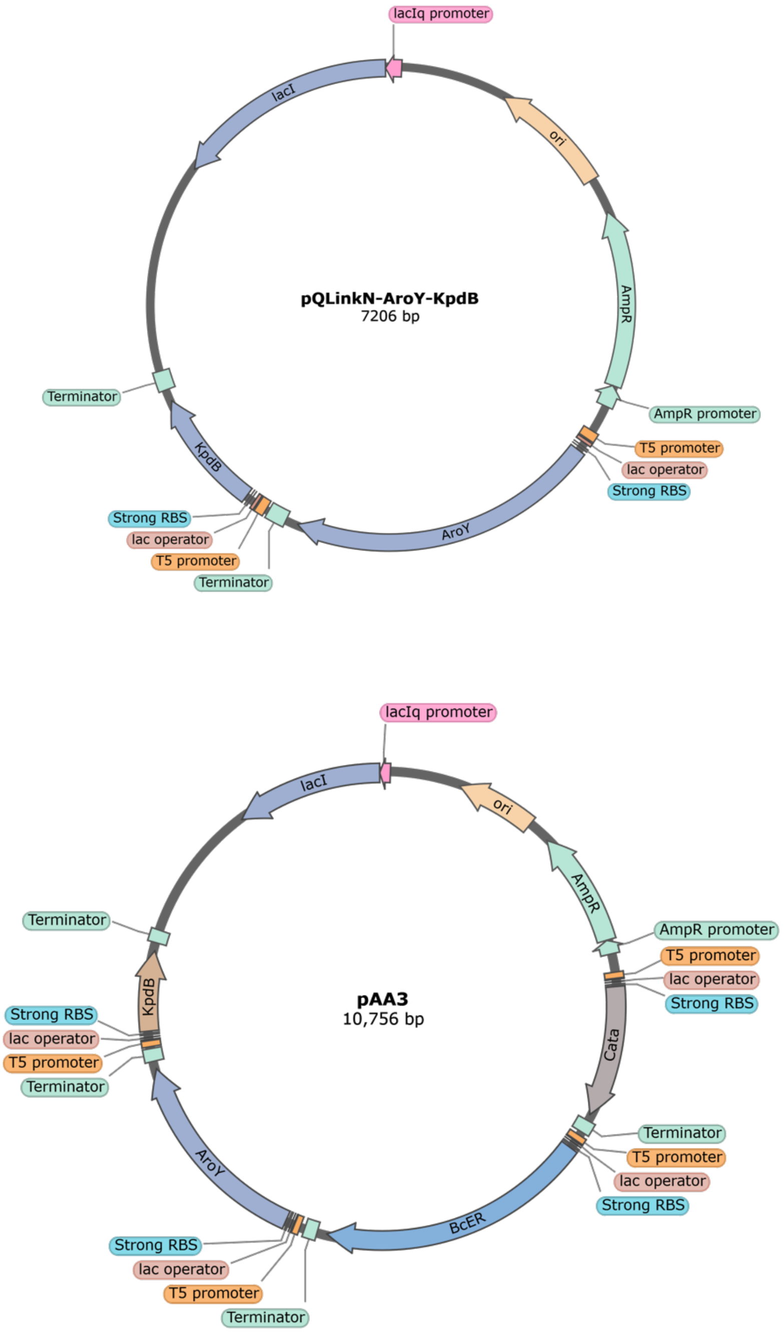

Figure S1. Plasmid maps of pQLinkN-*aroY-kpdB* and pAA3.

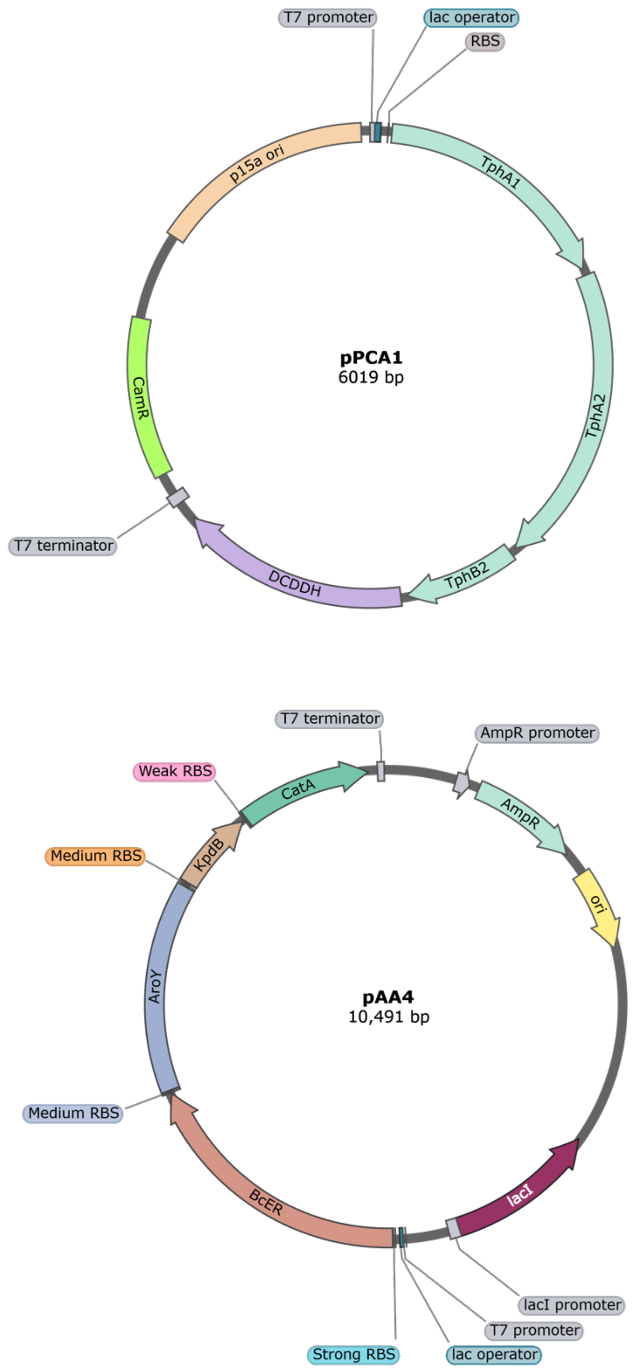

Figure S2. Plasmid maps of pPCA1 and pAA4.

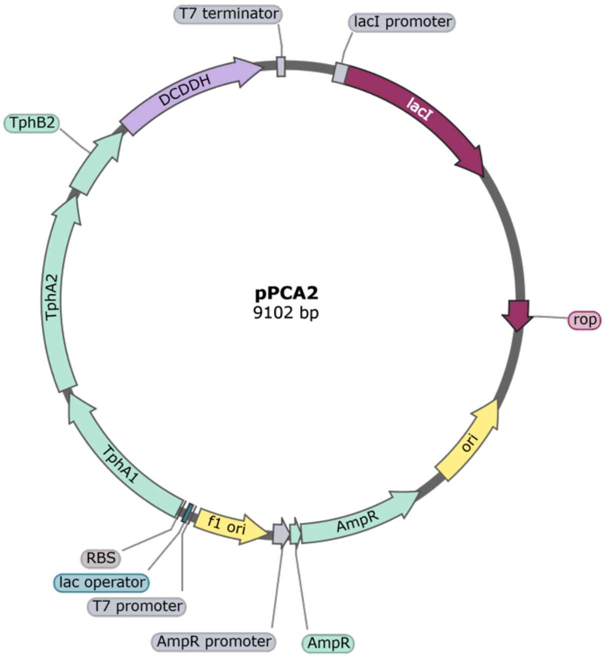

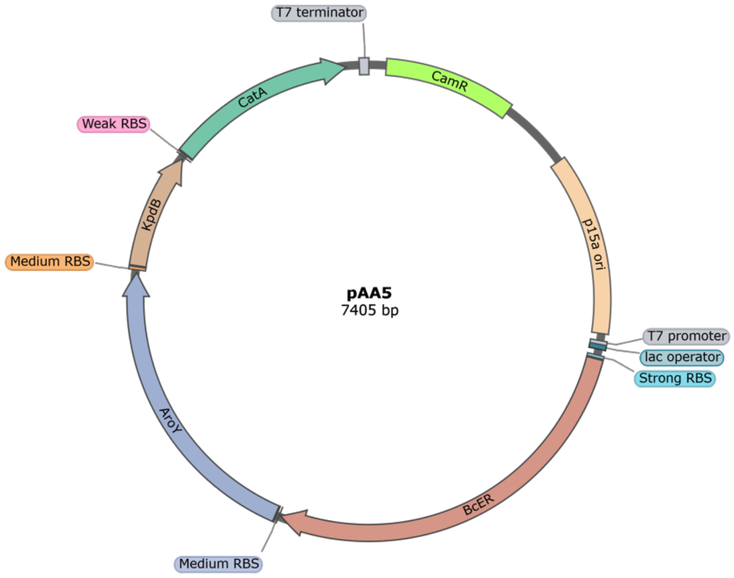

Figure S3. Plasmid maps of pPCA2 and pAA5.

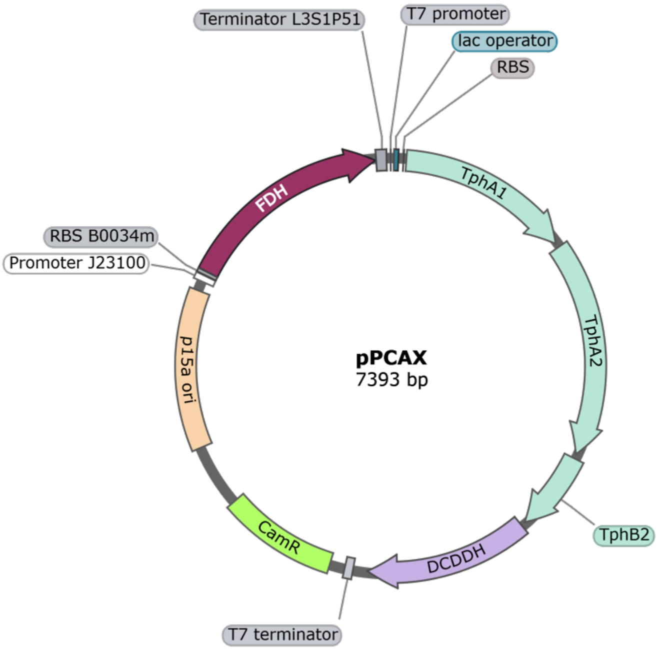

Figure S4. Plasmid maps of pPCAX-2 (with promoter J23100 for *fdh).*

S3 Genome Modification

Integration of the cassette from pPCA1 coding for *tphA1, tphA2, tphB2, dcddh* was achieved following the CRISPR-Cas9/λ-red method as reported by Bassalo et. al (2016). Donor plasmids were assembled via SLiCE cloning, combining plasmid pSS9 backbone (Addgene plasmid #71655) with locus-specific 600-bp homology arms amplified from *E. coli* BL21(DE3) genomic DNA, and the cassette amplified from pPCA1. The arabinose-inducible gRNA plasmid was generated by PCR and SLiCE assembly using the plasmid pSS9-gRNA as template DNA (Addgene #71656). Primers used are listed in Table S4.

Cassette integration in *E. coli* BL21(DE3) was performed by co-transforming the locus-specific gRNA, donor plasmid and pX2-Cas9 (Addgene plasmid #85811). After recovery at 37 °C for 4 h, positive transformants were selected on LB agar with ampicillin. Integration was confirmed by colony PCR using flanking (A, B) and cassette-specific primers (C) indicated in Table S5, using Phusion polymerase with High GC buffer. The CRISPR plasmids were cured by growing cells at 42 °C overnight without antibiotic selection and confirmed by loss of antibiotic resistance.

Table S4. Oligonucleotide primers used for construction of plasmid donor DNA for genome modification.

Plasmid	Template DNA	Sense	Sequence
pIS6_PCA	*E. coli* BL21(DE3) (5' homology arm)	F	gagtaaacttggtctgacagtgactactttccgcctga
		R	TTCCCCTATAGTGAGTCGTATTAaggatttcgtgtttctgcgtatcgt
	E. coli BL21(DE3) (3' homology arm)	F	GGTCTTGAGGGGTTTTTTGcttttaatggcgaatttctttattg
		R	gcttcaataatattgaaaaaggaagagtcatctggaaactgttgagtt
	pPCA1 (cassette)	F	acgcagaaacacgaaatcctTAATACGACTCACTATAGGGGAA
		R	cgatcaataaagaaattcgccattaaaagCAAAAAACCCCTCAAGACC
	pSS9 (backbone)	F	gaaactcaacagtttccagatgactcttcctttttcaatattattgaagc
		R	cgtcaggcggaaagtagtcactgtcagaccaagtttactc
pSS3_PCA	*E. coli* BL21(DE3) (5' homology arm)	F	gagtaaacttggtctgacagtgcgtgcaggttctgcaa
		R	TTCCCCTATAGTGAGTCGTATTAcatgctttgattgaccccgtatattacggg
	E. coli BL21(DE3) (3' homology arm)	F	GGTCTTGAGGGGTTTTTTGgtataaagggtattatttcttcgc
		R	gcttcaataatattgaaaaaggaagagtcattaataacgtaaataagaaagcc
	pPCA1 (cassette)	F	acggggtcaatcaaagcatgTAATACGACTCACTATAGGGGAA
		R	gcgaagaaataataccctttatacCAAAAAACCCCTCAAGACC
	pSS9 (backbone)	F	ggctttcttatttacgttattaatgactcttcctttttcaatattattgaagc
		R	gttgcagaacctgcacgcactgtcagaccaagtttactc
pSS9_PCA	*E. coli* BL21(DE3) (5' homology arm	F	gagtaaacttggtctgacagtgagaatacgccgaagttaaaatc
		R	TTCCCCTATAGTGAGTCGTATTAcaccgtttgacatatcacctgcgcca
	E. coli BL21(DE3) (5' homology arm	F	GGTCTTGAGGGGTTTTTTGatgttatatcgcgttgattattgatg
		R	gcttcaataatattgaaaaaggaagagtgcctacgattacgcatgg
	pPCA1 (cassette)	F	aggtgatatgtcaaacggtgTAATACGACTCACTATAGGGGAA
		R	agcatcaataatcaacgcgatataacatCAAAAAACCCCTCAAGACC
	pSS9 (backbone)	F	aagccatgcgtaatcgtaggcactcttcctttttcaatattattgaagc
		R	ttaacttcggcgtattctcactgtcagaccaagtttactc

pIS6 _gRNA	pSS9_gRNA	F	taatactagtcaacgatacgcagaaacacggttttagagctagaaatagc
		R	gctctaaaaccgtgtttctgcgtatcgttgactagtattatacctaggactg
pSS3 _gRNA	pSS9_gRNA	F	taatactagtcgtaatatacggggtcaatagttttagagctagaaatagc
		R	gctctaaaactattgaccccgtatattacgactagtattatacctaggactg
pSS9 _gRNA	pSS9_gRNA	F	taatactagtcgtaatatacggggtcaatagttttagagctagaaatagc
		R	gctctaaaactattgaccccgtatattacgactagtattatacctaggactg

Table S5. Oligonucleotide primers used for genotype confirmation of PCA-integration strains.

Template DNA	Sense	Sequence
SS3 locus integration candidates	A	tgcgtgcaggttctgcaa
	B	gacatcaaactgactgatctgg
	C	CTAACAAAGCCCGAAAGGAAG
IS6 locus integration candidates	A	tgactactttccgcctgac
	B	gactcaatggcatcagtgac
	C	CTAACAAAGCCCGAAAGGAAG
SS9 locus integration candidates	A	gtccggtaagaatcgtgg
	B	aacgcctgctcttattacgga
	C	CTAACAAAGCCCGAAAGGAAG

S4 Experimental Methods

S4.1 Protein expression in *E. coli* BL21(DE3)

A single colony of *E. coli* BL21(DE3) cells co-transformed with desired plasmids was used to inoculate 10 mL LB containing the appropriate antibiotics and cultures were incubated with orbital shaking at 37 °C overnight. Unless stated otherwise, protein expression cultures were prepared by inoculating 250 mL of LB media in a 500 mL baffled Erlenmeyer flask containing the appropriate antibiotics with 1% vol/vol overnight culture. The cultures were incubated with orbital shaking at 37 °C until OD_{600} = 0.45-0.55 and isopropyl β-D-1-thiogalactopyranoside (IPTG) was added to a final concentration of 0.4 mM. Cultures were incubated at 21 °C for 24 h before harvesting cells for biotransformation reactions.

S4.2 Whole cell biotransformation of terephthalic acid to adipic acid

Freshly prepared cells from expression cultures were harvested by centrifugation (4000 x*g*, 19 °C, 20 min) and the supernatant carefully removed. Cell pellets were resuspended in biotransformation buffer (M9- 3% glucose containing the appropriate substrate) to OD_{600}=60, unless otherwise noted. For biotransformation, a 3 mL aliquot of resuspended cells in reaction buffer was added to a 15 mL conical centrifuge tube per screening reaction. Reactions were incubated at 21 °C at 220 rpm for the required time and then analyzed by HPLC using the method outlined in Section S4.5. For increased aeration where necessary 8 holes were pierced into the lid of the reaction tube using a sterile needle.

S4.3 Fermentation reactions

Cultures of *E. coli* BL21(DE3) transformed with pPCA1 and pAA were grown to OD_{600} = 0.45–0.55 in either M9-glucose, M9-glycerol or LB and the appropriate antibiotics, and induced with IPTG (0.4 mM). After incubation at 21 ˚C and 220 rpm for 18 h, TA (5.0 mM) was added, and cultures were incubated for the required time before being analyzed by HPLC using the method outlined in Section S4.5.

S4.4 Bio-hydrogenation of *cis*,*cis*-muconic acid

A single colony of *E. coli* DD-2[11] was used to inoculate 10 mL LB containing ampicillin (100 µg/mL), chloramphenicol (30 µg/mL), and spectinomycin (25 µg/mL), and cultures were incubated with orbital shaking at 37 °C overnight. Protein expression cultures were prepared by inoculating 100 mL of M9CA media in a 500 mL Erlenmeyer flask containing ampicillin (100 µg/mL), chloramphenicol (30 µg/mL), and spectinomycin (25 µg/mL), with 1% v/v overnight culture. The cultures were incubated with orbital shaking at 37 °C until OD_{600} = 0.45–0.55, at which point IPTG and ammonium iron sulfate ($(NH_4)_2Fe(SO_4)_2$) were added to final concentrations of 0.5 mM and 50 µM, respectively. 5mL aliquots of culture were then transferred to Hungate tubes, sealed and sparged with nitrogen for 15 min and then incubated at 37 °C for 48 h.

Unless otherwise specified, 3.5 mL of biotransformation reaction filtered supernatant containing ccMA (under conditions described in Section S4.2) was mixed with 20 mol% of Pd catalyst (based on initial TA concentration) and transferred to hydrogen-producing cultures through rubber seals with an 18G, 1.5” needle. Cultures were incubated for a further 22h at 37 °C at 220 rpm before being analyzed by HPLC using the method outlined in Section S4.5.

S4.5 HPLC sample preparation from biotransformation reactions

A 200 µL sample was removed from the biotransformation reaction and quenched with 400 µL acetonitrile containing 0.15% v/v trifluoroacetic acid (TFA). Samples were vortexed for 10 sec and incubated at room temperature for 30 min before being vortexed for 10 sec and clarified by centrifugation (15000 x*g*, 10 min). A 300 µL aliquot of the supernatant was transferred to a fresh tube and the solvent allowed to evaporate in a fume cupboard overnight. 300 µL of MilliQ-H_2O containing 0.1% v/v TFA and 51 µM caffeine was added and the samples were clarified by centrifugation (15000 x*g*, 10 min) before analysis by HPLC using the method outlined in Section S1.

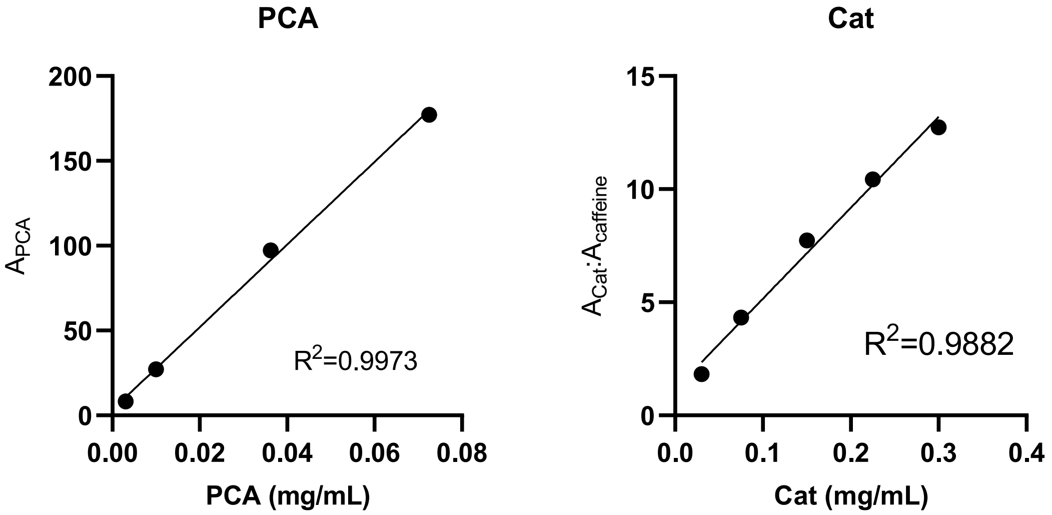

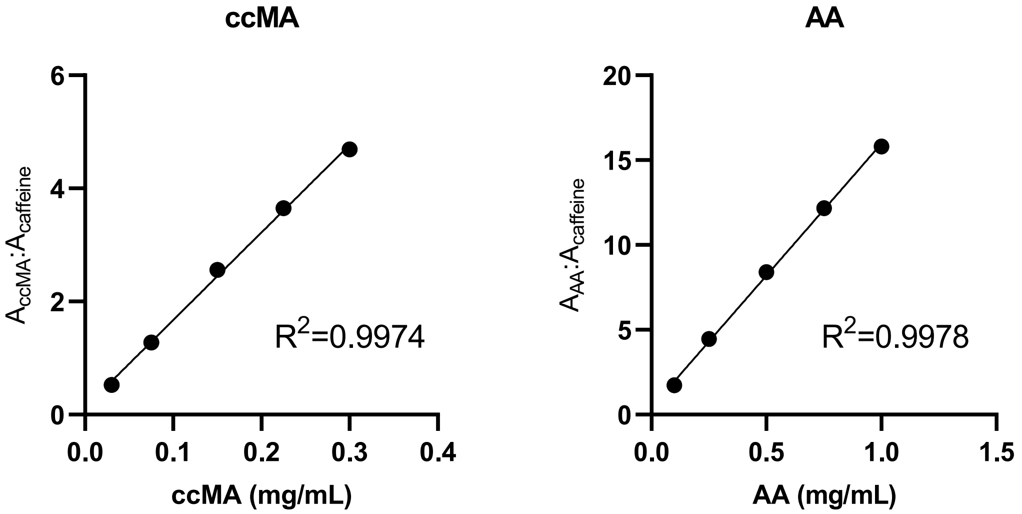

Figure S5. Response curves for pathway intermediates in comparison to a caffeine internal standard. Reaction samples were diluted to within the linear range prior to analysis by HPLC. Response curves generated using the mean values of triplicate runs for each analyte concentration.

S4.6 Cell immobilization in alginate hydrogels

Following the preparation of whole cell reactions as outlined in Section S4.2, cell samples were resuspended in 1.5% w/vol sodium alginate solution. The resulting solution was added dropwise to a 0.1 M aqueous solution of calcium chloride. The resulting beads were left to solidify for 10 min at room temperature. Alginate beads containing 1.5 mL cell samples were then added to 1.5 mL of M9 reaction buffer containing 2X TA before being incubated at 21 ˚C at 220 rpm for 24 h. Reaction supernatants were then analyzed by HPLC using the method outlined in Section S4.5.

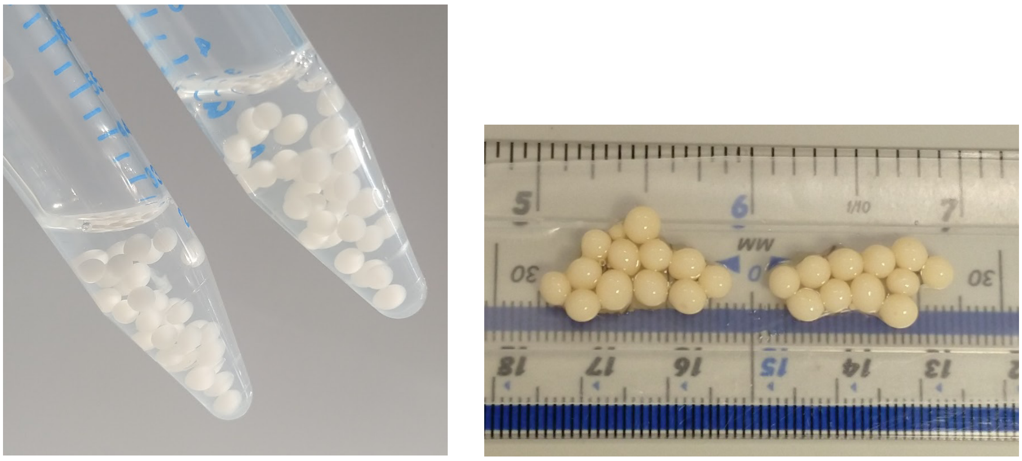

Figure S6. *E. coli* BL21(DE3) cells immobilized in calcium alginate beads (3–5 mm diameter).

S4.7 Terephthalic acid preparation from waste PET

PET Bottle: A waste PET plastic bottle was cleaned and cut into approximately 2 cm^2 segments. 1.8 g of PET fragments were then added to 180 mL of a solution containing 36 mL ethanol and 144 mL of 10% w/vol aqueous NaOH. The resulting mixture was stirred at 90 ˚C for 1 h before being filtered under vacuum. The filtrate was acidified using 50 mL of 37 % aqueous HCl solution, causing a white precipitate to form. The solid TA product was collected via vacuum filtration, analyzed by ^{1}H NMR (Section S5.6) and used without further purification in biotransformation reactions.

Stamping Foils: Following a similar procedure, 5 g of uncoated PET hot stamping foil waste samples (provided by API Foilmakers Ltd., UK) were cut into <10 cm^2 segments before being added to 36 mL ethanol and 144 mL of 10% w/vol aqueous NaOH (180 mL total vol) and stirred at 90 ˚C for 1 h. The resulting mixture was filtered under vacuum and acidified with 37% HCl to precipitate terephthalic acid, which was collected via vacuum filtration, analyzed by ^{1}H NMR (Section S5.6) and used without further purification in biotransformation reactions.

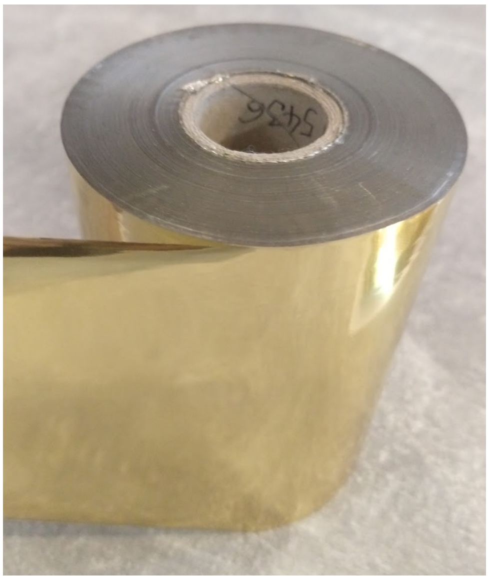

Figure S7. Gold colored stamping foil. HSF comprise a PET layer of *ca.* 10 microns thickness, in addition to layers of adhesives and pigments. A pure uncoated PET foil was used in this study.

S5 Supplementary data

S5.1 Protein expression

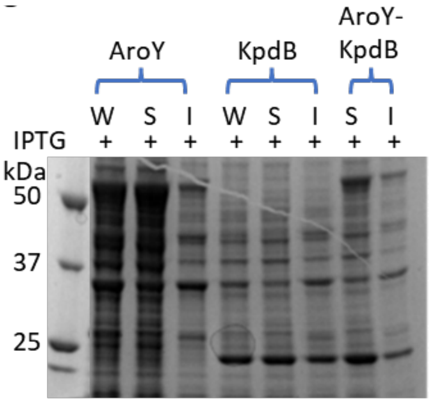

Figure S8. SDS-PAGE analysis of *E. coli* BL21(DE3) with either pQLinkN-*aroY*, pQLinkN-*kpdB*, or pQLinkN-*aroY*-*kpdB*. "W", "S", and "I" indicate whole cell lysate, soluble and insoluble fractions, respectively. Theoretical masses of target proteins (kDa): KpdB: 25; CatA: 35; AroY: 54; BcER: 75.

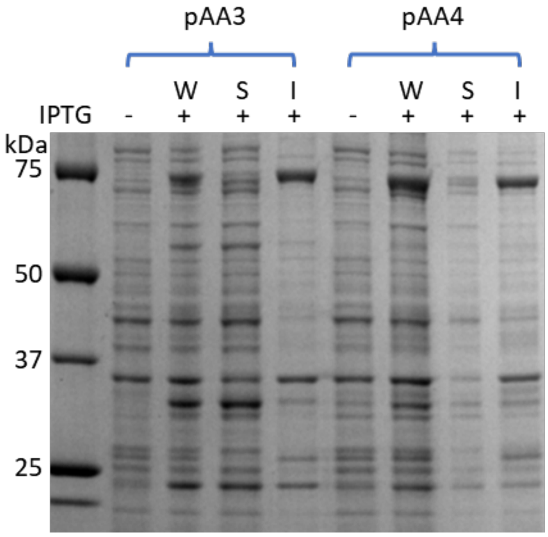

Figure S9. SDS PAGE analysis of *E. coli* BL21(DE3)_pAA3 and *E. coli* BL21(DE3)_pAA4 cells. "W", "S", and "I" indicate whole cell lysate, soluble and insoluble fractions respectively. Theoretical masses of target protein monomers (kDa): KpdB: 25; CatA: 35; AroY: 54; BcER: 75.

S5.2 Whole-cell mixing experiments

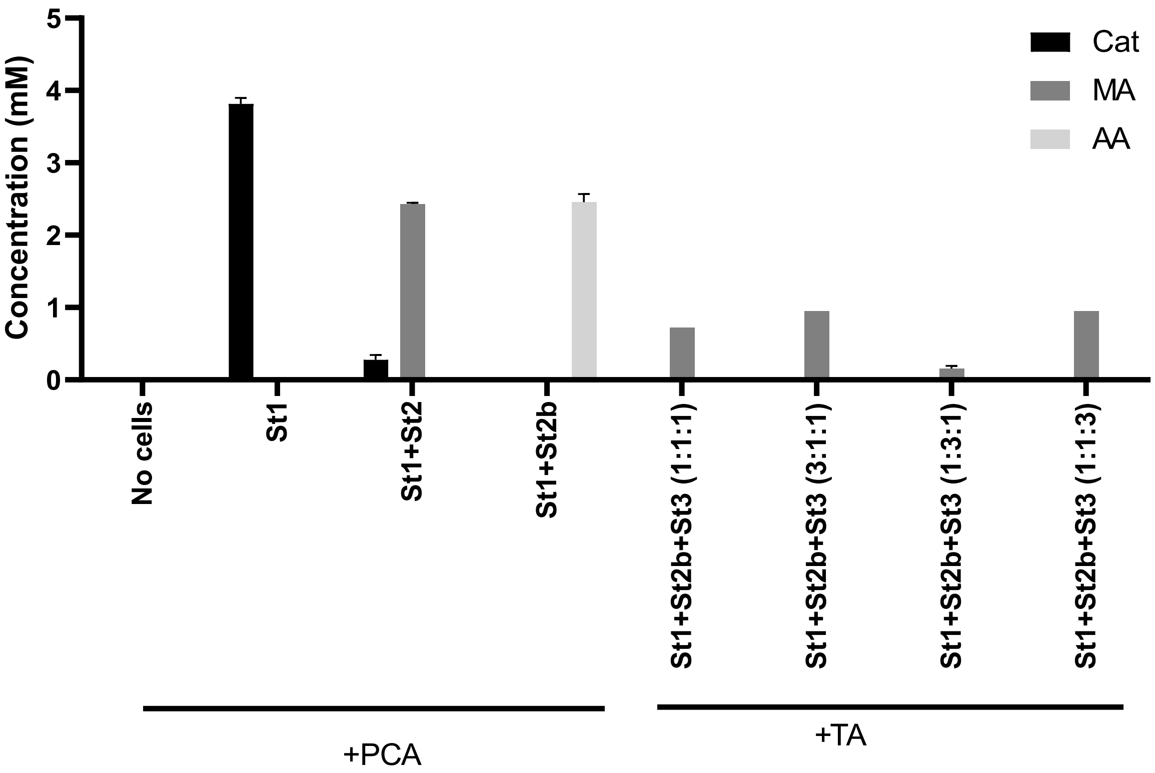

Figure S10. Initial whole-cell mixing experiments for TA upcycling to AA. All reactions performed at OD_{600}=122 with equal amounts of all strains (n=3); reactions mixing three strains (n=1).

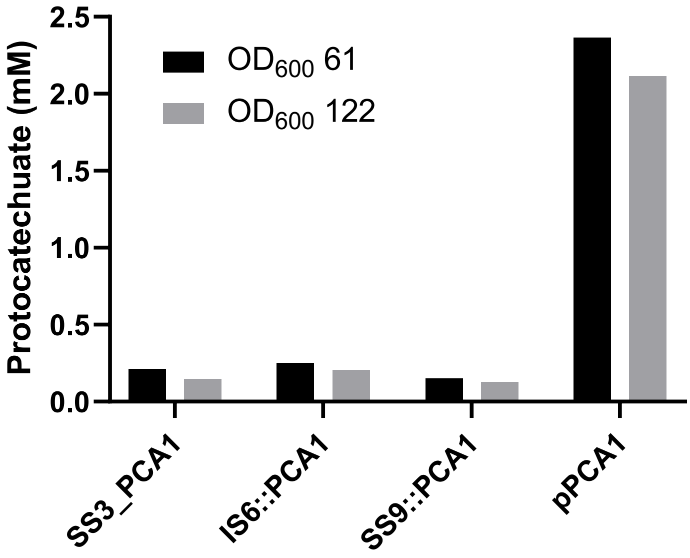

Figure S11. Production of ccMA from various *E. coli* BL21(DE3) strains, expressing TPADO from a chromosomal integration at different loci or from plasmid pPCA1. For each strain to cell concentrations were tested (n = 1).

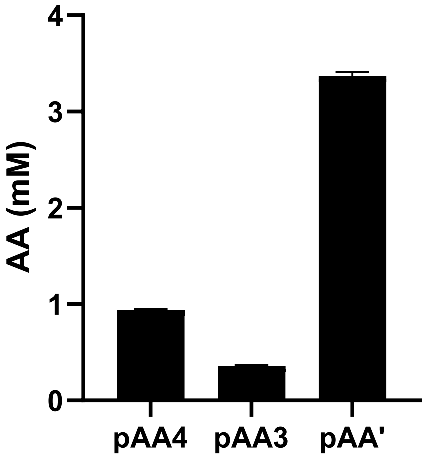

Figure S12. Reduction of 5 mM ccMA from engineered *E. coli* BL21(DE3) harboring pAA3 or pAA4, or pAA' (pQlinkN-*gcoA*/*gcoB*/*catA*/*BcER*) used as control[1]. Triethanolamine (100 mM) was added to each reaction to solubilize ccMA.

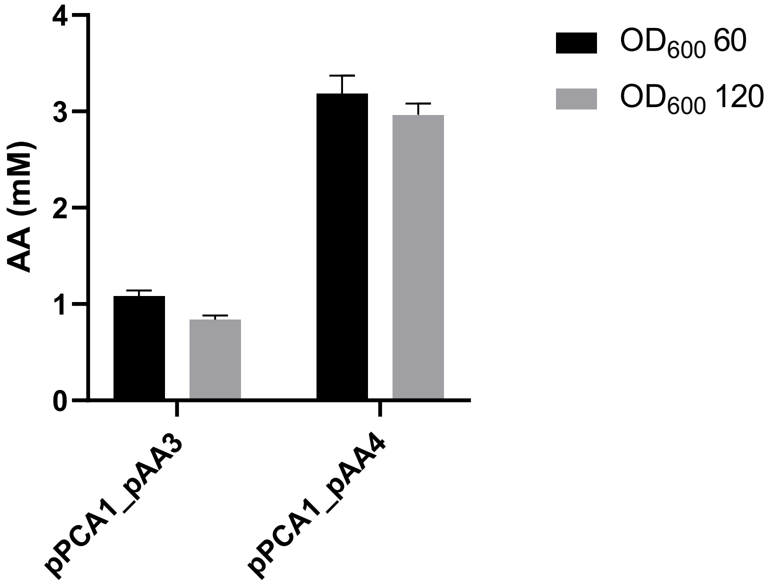

Figure S13. Bio-reduction of ccMA by engineered *E. coli* cells harboring pAA4 and pAA3. Reactions were performed at two different cell concentrations with n=3 for reactions at OD_{600}=60 and n=2 for reactions at OD_{600}=120.

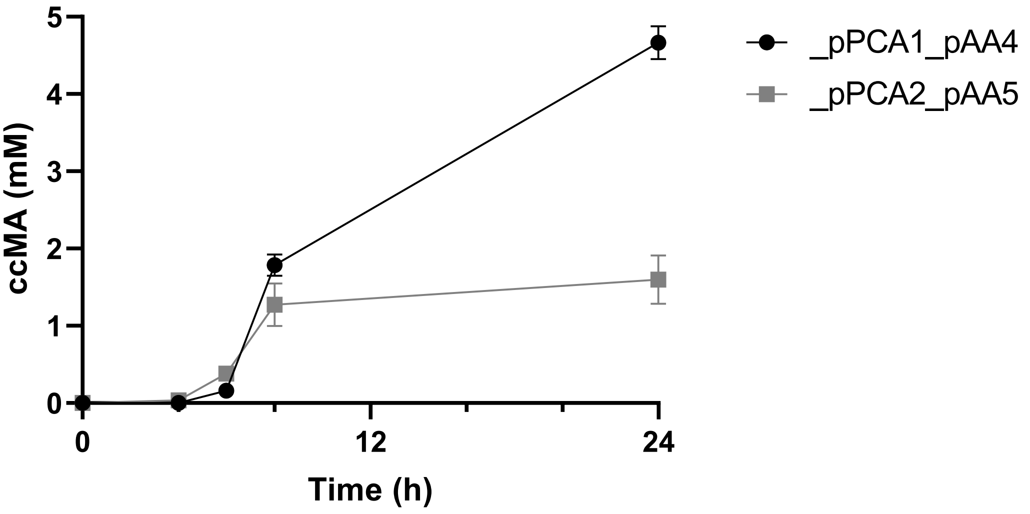

Figure S14. Kinetic analysis of *E. coli* BL21(DE3) harboring plasmids pPCA1 and pAA4 or pPCA2 and pAA5. Reactions performed in duplicates (n=2).

S5.3 Expression optimization and reaction optimization

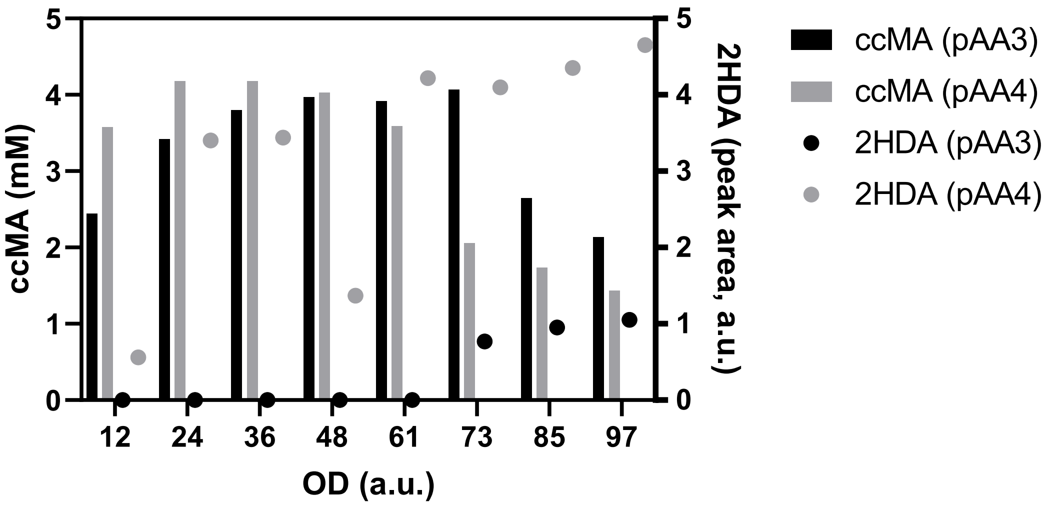

Figure S15. Effect of cell density on ccMA and 2HDA production from TA (5 mM). *E. coli* BL21(DE3)_pPCA1_pAA3 or *E. coli* BL21(DE3)_pPCA1_pAA4 strains were used.

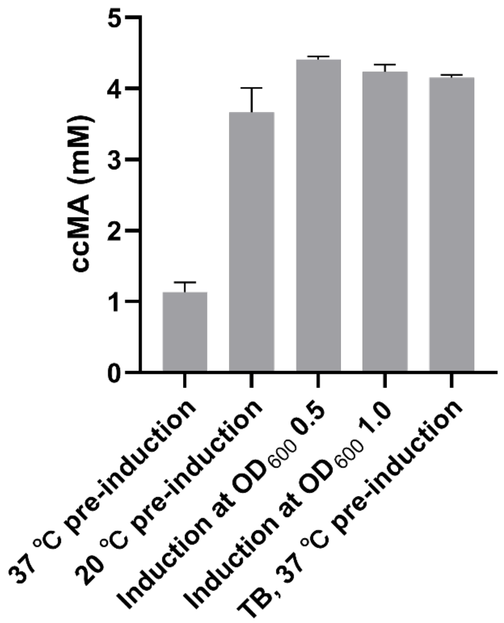

Figure S16. Altering growth and protein expression conditions*. E. coli* BL21(DE3)_pPCA1_pAA3 and 5 mM TA was used in all experiments. Methodology followed that described in Section S4.2.

S5.4 Biocompatible hydrogenation

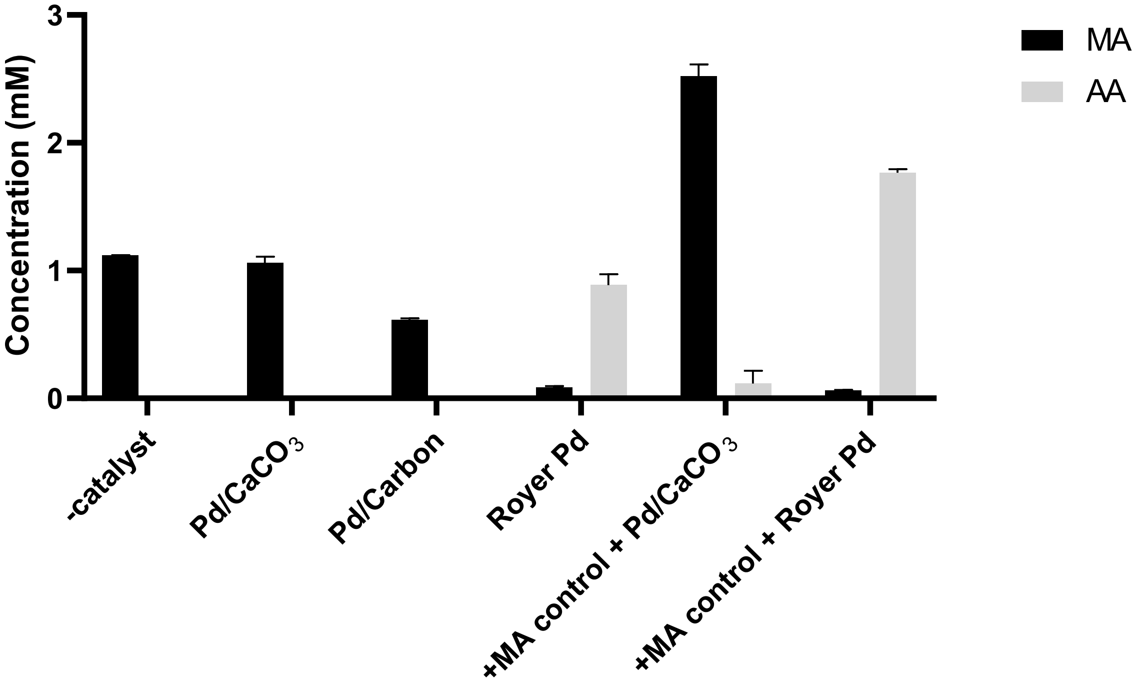

Figure S17. Bio-hydrogenation of metabolic *cis,cis*-muconic acid. Spent *E. coli* BL21(DE3)_pPCA1_pAA4 biotransformation reactions and Pd catalysts were added to cultures of *E. coli* DD-2. Control reactions contained exogenous ccMA dissolved in M9 media.

S5.5 Cell immobilization experiments

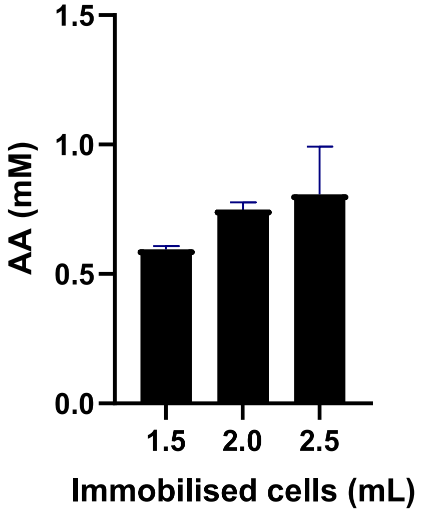

Figure S18. AA synthesis from TA using immobilized *E. coli*_BL21(DE3)pPCA1_pAA4 cells at various alginate cell volume.

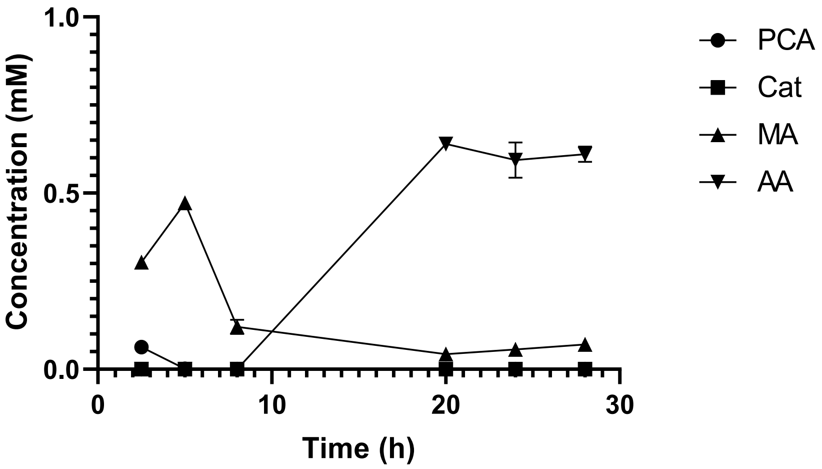

Figure S19. Reaction time-course experiment using alg-*E. coli*_pPCA1_pAA4 cells. Metabolite concentrations were determined by HPLC according to the method outlined in Section S4.5.

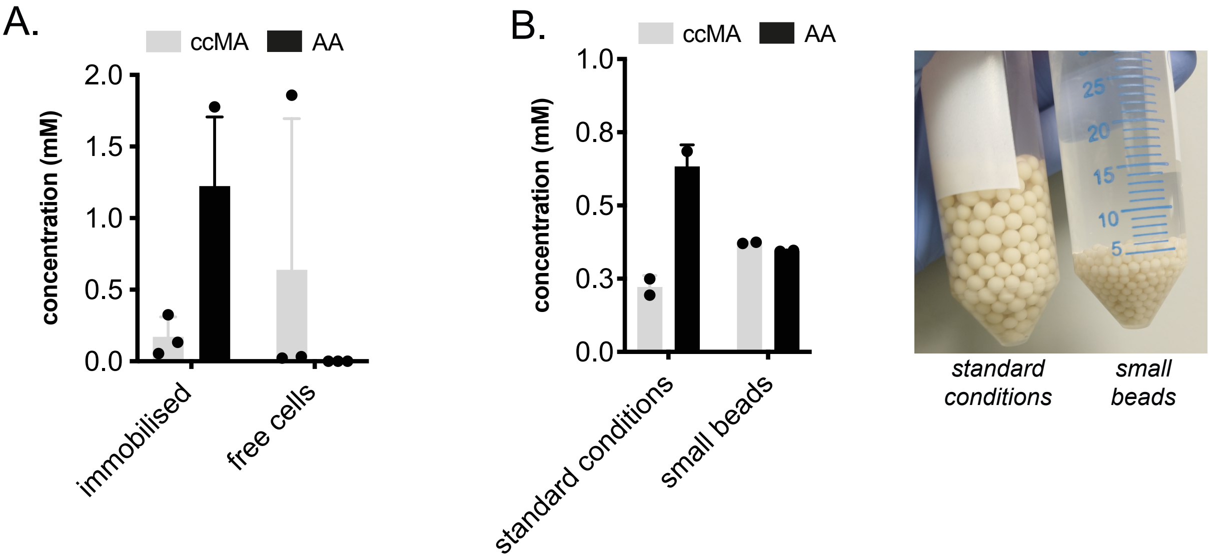

Figure S20. Control reactions showing increased BcER activity in alginate beads. A) reactions run with 2.5 mM ccMA for 24 h in aerobic conditions after 6 h of pre incubation without substrate. B) reactions run with 2.5 mM TA in various sizes of alginate beads.

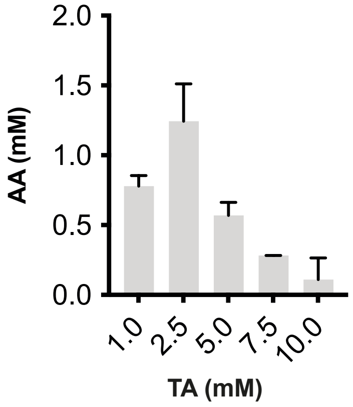

Figure S21. Whole-cell biotransformation using increasing concentrations of terephthalic acid.

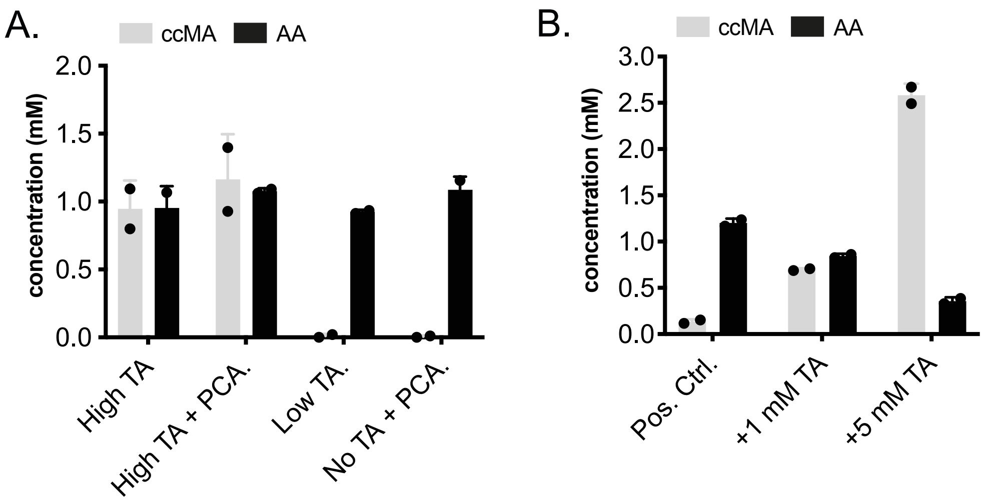

Figure S22. Control reactions showing terephthalate dependent BcER inhibition. A) ccMA and AA production from 1 mM PCA, 1 mM TA (Low TA), 1 mM PCA or 5 mM TA (High TA). B) AA production from 2.5 mM ccMA after the addition of TA.

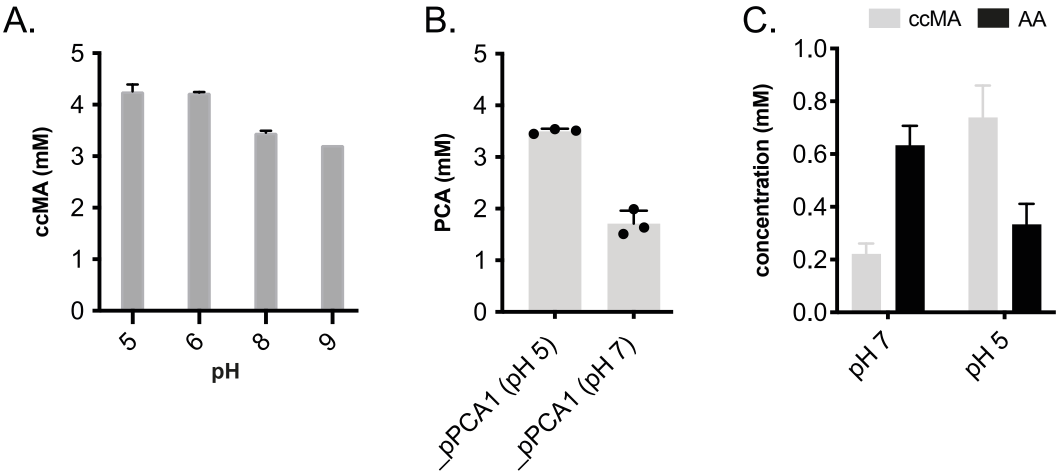

Figure S23. Effect of pH on whole pathway and PCA synthesis. A) Conversion of 5 mM ccMA by non-immobilized _pPCA1_pAA4 cells at various starting pH values. B) Effect of pH on conversion of 5 mM TA to PCA after 5 h. C) Synthesis of ccMA and AA from TA in immobilized *E. coli*_pPCA1_pAA4 cells at different pH values.

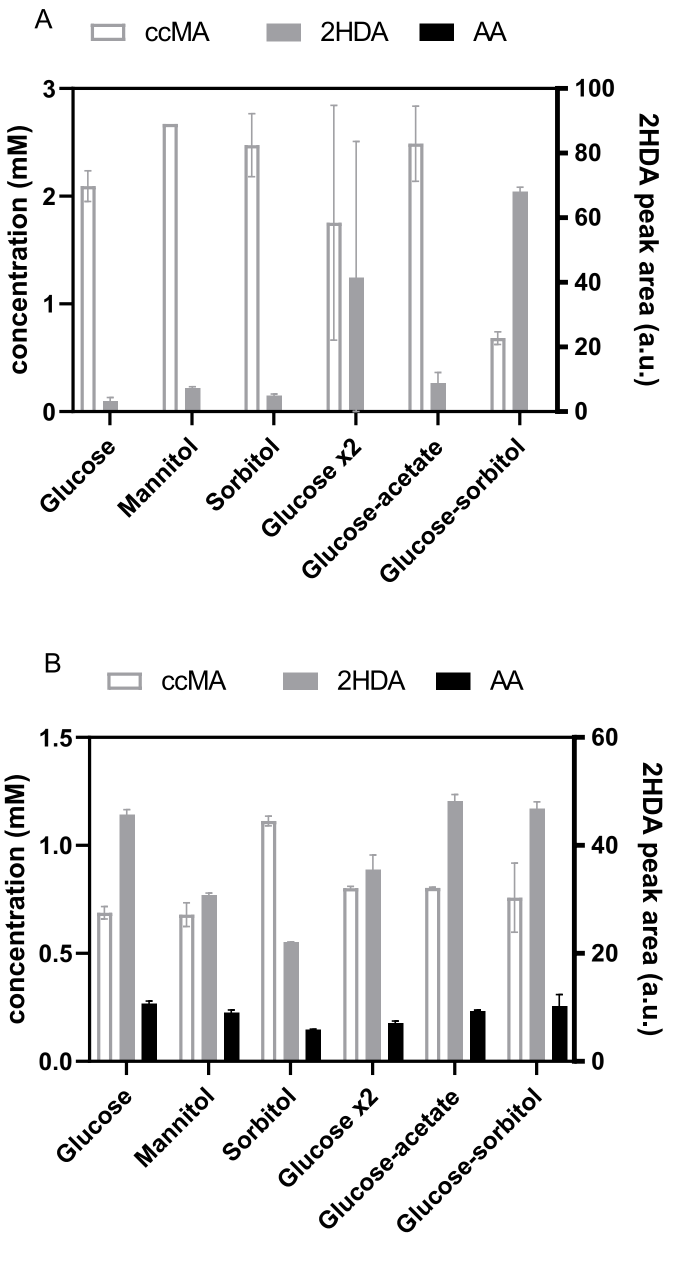

Figure S24. Effect of carbon source on the whole-cell biotransformation of 2.5 mM TA to ccMA and AA. A) Biotransformation using *E. coli*_pPCA1_pAA4. B) Biotransformation using immobilized alg-*E. coli*_pPCA1_pAA4 cells. Each carbon source added at 83 mM (equivalent to 1.5% w/v for glucose). Sodium acetate added at 167 mM.

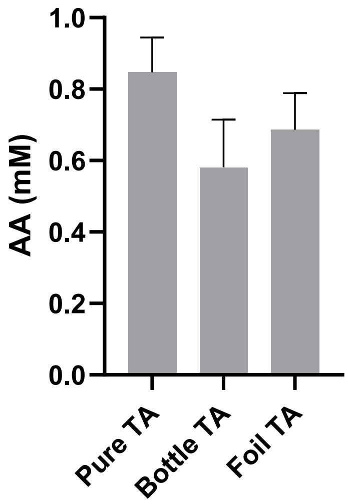

Figure S25. Whole-cell biotransformation experiments using commercial TA, and TA isolated from various PET sources, as outlined in Section S4.7.

S5.6 NMR Spectroscopy

Standards of TA, ethylene glycol, *bis*(2-hydroxyethyl)terephthalate (BHET), and disodium terephthalate were compared to TA isolated from hydrolyzed PET bottles or stamping foils.

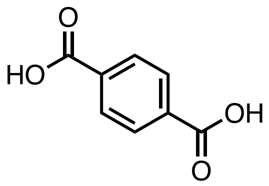

^{1}H NMR (600 MHz, D_2O) δ/ppm: 7.87-7.85 (m, 4H); ^{13}C NMR (600MHz, D_2O) δ: 175.2, 138.6, 128.6, 128.5, 128.4.

For quantification of TA, 1.2 mg of TA isolated from a hydrolyzed PET bottle or 2 mg TA isolated from a hydrolyzed PET stamping foil was dissolved in D_2O containing 5mM 1,3,5-trimethoxybenzene as an internal standard.

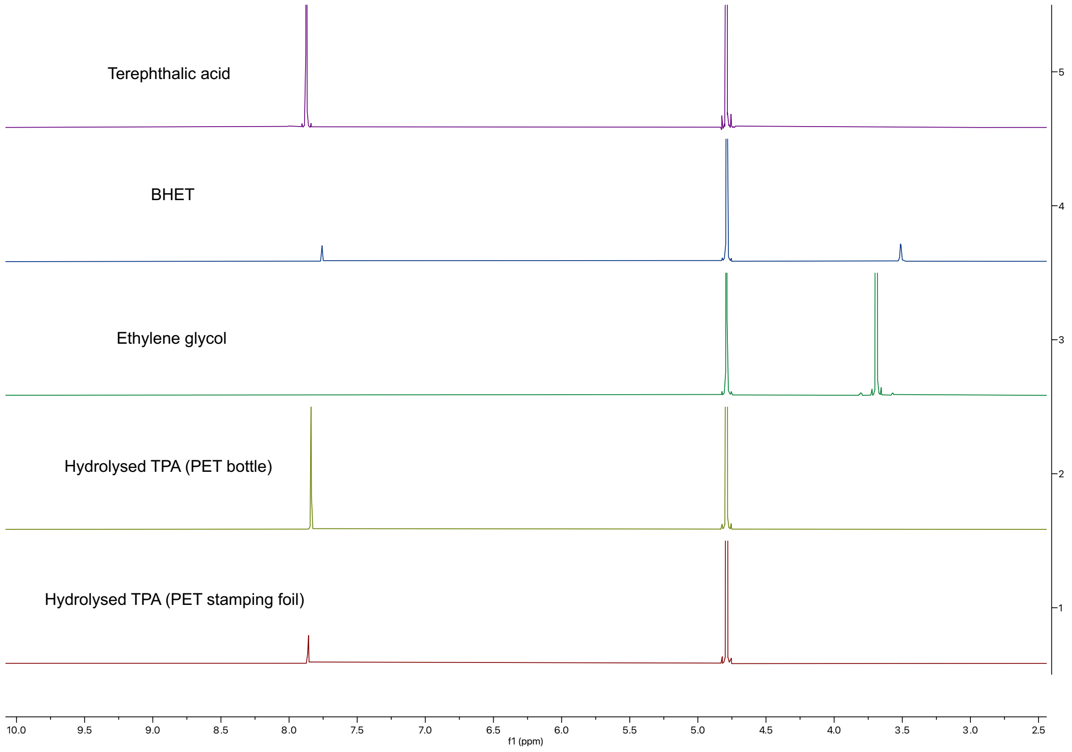

Figure S26. ^{1}H NMR spectra of TA, BHET, and ethylene glycol commercial standards, aligned with TA obtained by hydrolysis of either a plastic bottle or hot stamping foil.

S6 References

1. Suitor, J. T.; Varzandeh, S.; Wallace, S., One-Pot Synthesis of Adipic Acid from Guaiacol in Escherichia coli. *ACS Synthetic Biology* **2020,** *9* (9), 2472-2476.
2. Cohen, S. N.; Chang, A. C.; Hsu, L., Nonchromosomal antibiotic resistance in bacteria: genetic transformation of Escherichia coli by R-factor DNA. *Proceedings of the National Academy of Sciences of the United States of America* **1972,** *69* (8), 2110-2114.
3. Motohashi, K., A simple and efficient seamless DNA cloning method using SLiCE from Escherichia coli laboratory strains and its application to SLiP site-directed mutagenesis. *BMC Biotechnology* **2015,** *15* (1), 47.
4. Aslanidis, C.; De Jong, P. J., Ligation-independent cloning of PCR products (LIC-PCR). *Nucleic acids research* **1990,** *18* (20), 6069-6074.
5. Beyer, H. M.; Gonschorek, P.; Samodelov, S. L.; Meier, M.; Weber, W.; Zurbriggen, M. D., AQUA Cloning: A Versatile and Simple Enzyme-Free Cloning Approach. *PLOS ONE* **2015,** *10* (9), e0137652.
6. Zhang, Y.; Werling, U.; Edelmann, W., Seamless Ligation Cloning Extract (SLiCE) Cloning Method. In *DNA Cloning and Assembly Methods*, Valla, S.; Lale, R., Eds. Humana Press: Totowa, NJ, 2014; pp 235-244.
7. Valenzuela-Ortega, M.; French, C., Joint universal modular plasmids (JUMP): a flexible vector platform for synthetic biology. *Synth Biol (Oxf)* **2021,** *6* (1), ysab003.
8. Sadler, J. C.; Wallace, S., Microbial synthesis of vanillin from waste poly(ethylene terephthalate). *Green Chemistry* **2021,** *23* (13), 4665-4672.
9. Bassalo, M. C.; Garst, A. D.; Halweg-Edwards, A. L.; Grau, W. C.; Domaille, D. W.; Mutalik, V. K.; Arkin, A. P.; Gill, R. T., Rapid and Efficient One-Step Metabolic Pathway Integration in E. coli. *ACS Synthetic Biology* **2016,** *5* (7), 561-568.
10. Scheich, C.; Kümmel, D.; Soumailakakis, D.; Heinemann, U.; Büssow, K., Vectors for co-expression of an unrestricted number of proteins. *Nucleic Acids Research* **2007,** *35* (6), e43-e43.
11. Agapakis, C. M.; Ducat, D. C.; Boyle, P. M.; Wintermute, E. H.; Way, J. C.; Silver, P. A., Insulation of a synthetic hydrogen metabolism circuit in bacteria. *Journal of biological engineering* **2010,** *4*, 3.
